# Stable Neural Population Dynamics in the Regression Subspace for Continuous and Categorical Task Parameters in Monkeys

**DOI:** 10.1523/ENEURO.0016-23.2023

**Published:** 2023-07-07

**Authors:** He Chen, Jun Kunimatsu, Tomomichi Oya, Yuri Imaizumi, Yukiko Hori, Masayuki Matsumoto, Takafumi Minamimoto, Yuji Naya, Hiroshi Yamada

**Affiliations:** 1 School of Psychological and Cognitive Sciences, Peking University, Beijing 100805, People’s Republic of China; 2 Division of Biomedical Science, Institute of Medicine, University of Tsukuba, Tsukuba 305-8577, Japan; 3 Transborder Medical Research Center, University of Tsukuba, Tsukuba 305-8577, Japan; 4 The Brain and Mind Institute, University of Western Ontario, London N6A 3K7, Canada; 5 Department of Physiology and Pharmacology, University of Western Ontario, London N6A 3K7, Canada; 6 Medical Sciences, University of Tsukuba, Tsukuba 305-8577, Japan; 7 Department of Functional Brain Imaging, National Institutes for Quantum Science and Technology, Chiba 263-8555, Japan; 8 IDG/McGovern Institute for Brain Research at Peking University, Beijing 100805, People’s Republic of China; 9 Beijing Key Laboratory of Behavior and Mental Health, Peking University, Beijing 100805, People’s Republic of China

**Keywords:** dimensional reduction, monkey, neural population dynamics, regression subspace

## Abstract

Neural population dynamics provide a key computational framework for understanding information processing in the sensory, cognitive, and motor functions of the brain. They systematically depict complex neural population activity, dominated by strong temporal dynamics as trajectory geometry in a low-dimensional neural space. However, neural population dynamics are poorly related to the conventional analytical framework of single-neuron activity, the rate-coding regime that analyzes firing rate modulations using task parameters. To link the rate-coding and dynamic models, we developed a variant of state-space analysis in the regression subspace, which describes the temporal structures of neural modulations using continuous and categorical task parameters. In macaque monkeys, using two neural population datasets containing either of two standard task parameters, continuous and categorical, we revealed that neural modulation structures are reliably captured by these task parameters in the regression subspace as trajectory geometry in a lower dimension. Furthermore, we combined the classical optimal-stimulus response analysis (usually used in rate-coding analysis) with the dynamic model and found that the most prominent modulation dynamics in the lower dimension were derived from these optimal responses. Using those analyses, we successfully extracted geometries for both task parameters that formed a straight geometry, suggesting that their functional relevance is characterized as a unidimensional feature in their neural modulation dynamics. Collectively, our approach bridges neural modulation in the rate-coding model and the dynamic system, and provides researchers with a significant advantage in exploring the temporal structure of neural modulations for pre-existing datasets.

## Significance Statement

Our results differ from earlier studies and suggest that our state-space analysis in the regression subspace provides a mechanistic neural population structure for visual recognition of items when monkeys perceived continuous and categorical task parameters. The neural population dynamics obtained from different brain regions using different behavioral tasks were similar and may share some common underlying information processing in a neural network. Our approach provides a simple framework for incorporating the single-neuron approach into the dynamic model as a procedure for describing neural modulation dynamics in the brain. This analytic extension gives researchers a significant advantage in that all types of pre-existing data for single-neuron activity are useful for easily exploring their dynamics in a low-dimensional neural modulation space.

## Introduction

Recent innovations in state-space analyses have provided deep insights into the dynamic structure of information processing in neural population activity (Brendel et al., 2011; Churchland et al., 2012; Mante et al., 2013). The identified fine temporal structures are known as neural population dynamics and are considered to reflect underlying computations occurring in a neural network in the sensory, cognitive, and motor domains (Churchland et al., 2012; Raposo et al., 2014; Murray et al., 2017; Aoi et al., 2020; Osako et al., 2021; Rossi-Pool et al., 2021). Neural population dynamics provide a different perspective from conventional analytical frameworks such as the rate-coding model, which usually analyzes neural modulations by task parameters. However, dynamic and conventional rate-coding models have rarely been compared with analyze neural activity. Thus, an important question remains regarding how these two approaches reflect putatively different or shared aspects of information processing used by neurons in the underlying neuronal network.

An early analytical framework for information processing was developed to describe the functional role of separately recorded single-neuron activity (Mountcastle and Henneman, 1949; Hubel and Wiesel, 1959; Evarts, 1968; Wurtz, 1968), mostly using the rate-coding model (Dayan and Abbott, 2001). Neuronal discharge rates were assumed to be modulated by mathematical parameters controlled in an experimental task. Examples of these task parameters are listed, such as a Gabor function in the visual cortex (Tolhurst and Movshon, 1975; Jones and Palmer, 1987), movement direction and muscle force in the motor cortex (Fetz and Cheney, 1980; Georgopoulos et al., 1982), reward value in the parietal cortex (Platt and Glimcher, 1999), and spatial awareness during navigation in the hippocampus (HPC; O’Keefe and Dostrovsky, 1971). Recently, large-scale multichannel recording technology (Buzsáki et al., 2015; Jun et al., 2017) has been developed to understand information processing used by neural networks. It has enabled the observation of single-neuron activities in the tens of thousands, reinforcing the need for a novel theoretical framework for neural computations (Yuste, 2015), one of which is neural population dynamics provided by state-space analysis (Timothy and Bona, 1968). State-space analysis has emerged with a greater focus on temporal changes in neuronal activity combined with dimensional reduction techniques (Elsayed and Cunningham, 2017; Aoi and Pillow, 2018; Keemink and Machens, 2019; Saxena and Cunningham, 2019; Vyas et al., 2020). Both analytical frameworks have described brain functions in various functional domains. However, the relationship between the dynamic and conventional rate-coding models remains poorly understood.

Some remarkable studies have attempted to find the link between the rate-coding and dynamic models. For example, demixed principal component analysis (dPCA; Brendel et al., 2011; Kobak et al., 2016) decomposed neural population data into latent components related to discrete task parameters, such as stimulus and binary choices. In another approach, targeted dimensionality reduction (TDR) analysis (Mante et al., 2013; Aoi et al., 2020) incorporated linear regression into neural population dynamics with respect to task parameters, such as the stimulus, binary choice, and task context, and identified the encoding axis of these task parameters in a multidimensional neural space. These state-of-the-art techniques visualize complex datasets with multiple task parameters and depict the temporal structures of task-related neural modulation. However, they have several limitations when applied to actual data and require the user to precisely understand these elaborate procedures involving complex multistep mathematical processes (Kobak et al., 2016; Aoi et al., 2020). Although these analytical tools describe the temporal structure of neural modulation as a latent component in the multidimensional neural space (Kobak et al., 2016, their Fig. 3B; Aoi et al., 2020, their Fig. 3A) the extracted modulation structures do not simply correspond to the results from the rate-coding analyses. Thus, it is worthwhile to develop a simple, user-friendly method to extract temporal structures of neural modulations that are compatible with the rate-coding model.

We previously developed a variant of state-space analysis for continuous parameters (Yamada et al., 2021) that describes how a neuronal population dynamically represents some cognitive task parameters in a regression subspace. This analysis simply extracts the temporal structures of neural modulations in the following two steps using standard statistical software: (1) an estimation of regression coefficients for neural modulations by task parameters across time and neurons, which provides a regression matrix representing the extent of neural modulation as a function of time in a neural population; and (2) application of PCA to the regression matrix, which provides neural dynamics in the regression subspace (i.e., temporal structures of neural modulations by the task parameters). Our previous study successfully captured neural modulation dynamics using continuous task parameters related to value-based decision-making. However, the analysis was only performed for continuous task parameters, and it is still unclear whether this approach could be expanded to neural population activity modulated by noncontinuous categorical task parameters.

**Figure 1.**
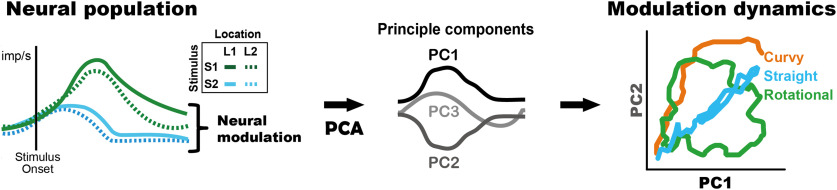
Schematic depictions of the state-space analysis in the regression subspace. The state-space analysis in the regression subspace provides the neural dynamics for activity modulations. In a particular experimental condition, neural population activity composed of multiple neurons is modulated by the task variables [e.g., location (L1, L2) and stimulus (S1, S2; left)]. By applying PCA (principal component analysis) for neural modulations (left), predominant components of the neural modulations are extracted (middle) and depicted as the neural trajectory (right). Typical examples of neural population structures observed previously are straight, curvy, and rotational structures. imp/s indicates impulse per second. PC1 to 3 indicates first to third principle components, respectively.

In the present study, we developed an analysis method that could be applied to existing datasets using a typical factorial design for conventional single-neuron recordings (Fig. 1), that is, categorical task parameters, from the hippocampus of monkeys while memorizing a visual item and its location (Chen and Naya, 2020). Thereafter, we compared neural modulation dynamics between continuous and categorical task parameters for visual stimulus modulations. In particular, we have described neural dynamics within a short time period of 0.6 s when a visual cue appears, and neurons encode the information of the categorical or continuous task parameter conveyed by the visual stimulus. This approach successfully provided the temporal structures of neural modulations for both types of task parameters as trajectory geometry in the low-dimensional neural modulation space. The extracted geometries for both task parameters form a straight geometry, suggesting that their functional relevance is characterized as a unidimensional feature in their neural modulation dynamics. Thus, analyzing the neural modulation dynamics for all types of pre-existing data is beneficial, allowing researchers to incorporate the rate-coding model into a dynamic system.

## Materials and Methods

### Subjects and experimental procedures

Four macaque monkeys were used in two experiments [Experiment 1 (Exp. 1): monkey SUN, *Macaca mulatta,* 7.1 kg, male; monkey FU, *Macaca fuscata*, 6.7 kg, female; Exp. 2: monkey A, *M. mulatta,* 9.3 kg, male; monkey D, *M. mulatta,* 9.5 kg, male). All experimental procedures were approved by the Animal Care and Use Committee of the University of Tsukuba (Exp. 1, protocol #H30.336), and the institutional animal care and use of laboratory animals, approved by Peking University (Exp. 2, project #Psych-YujiNaya-1). All procedures were performed in compliance with the US Public Health Service *Guide for the Care and Use of Laboratory Animals*.

### Behavioral task

#### Cued lottery tasks in Experiment 1

The animals performed one of two visually cued lottery tasks: a single-cue or choice task. Neuronal activity was only recorded during the single-cue task.

The animals performed the task under dim lighting conditions in an electromagnetically shielded room. Eye movements were measured using a video camera system at 120 Hz (EyeLink, SR Research). Visual stimuli were generated by a liquid crystal display at 60 Hz placed 38 cm from the face of the monkey when seated. At the beginning of the single-cue task trials, the monkeys had 2 s to align their gaze within 3° of a 1° diameter gray central fixation target. After fixation for 1 s, a pie chart was presented for 2.5 s to provide information regarding the probability and magnitude of rewards in the same location as the central fixation target. The probability and magnitude of the rewards were associated with the number of blue and green 8° pie chart segments, ranging from 0.1 to 1.0 ml in 0.1 ml increments for magnitude, and from 0.1 to 1.0 in 0.1 increments for probability. Following a 0.2 s interval from the removal of the pie chart, either a 1 or a 0.1 kHz tone of 0.15 s duration was provided to indicate reward or no-reward outcomes, respectively. After a 0.2 s interval following the high tone, a fluid reward was delivered, whereas no rewards were delivered following the low tone. An intertrial interval of 4–6 s was used. During the choice task, the animals were instructed to choose one of two peripheral pie charts, each of which indicated either the probability or magnitude of an upcoming reward. The two target options were presented for 2.5 s at 8° to the left or right of the central fixation location. The animals received a fluid reward, indicated by the green pie chart of the chosen target, with the probability indicated by the blue pie chart. Otherwise, no reward was delivered.

A total of 100 pie charts were used in the experiments, which were composed of 10 levels of probability and magnitude of rewards. In the single-cue task, the 100 pie charts were presented once in random order. In the choice task, two pie charts were randomly allocated to the two options. During one session of electrophysiological recording, ∼30–60 trial blocks of the choice task were interleaved with 100–120 trial blocks of the single-cue task.

#### Item-location-retention task in Experiment 2

The animals performed the task under dim light conditions in an electromagnetically shielded room. The task started with an encoding phase, which was initiated by the animal pulling a lever and fixating on a white square (0.6°) presented within one of four quadrants at 12.5° (monkey A) or 10° (monkey D) from the center of the touch screen (17 inches; MicroTouch Display M1700SS, 3M), situated ∼28 cm from the subjects. Eye position was monitored using an infrared digital camera with a sampling frequency of 120 Hz (model ETL-200, ISCAN). After fixation for 0.6 s, one of the six items (radius: 3.0° for monkey A; 2.5° for monkey D) was presented in the same quadrant as a sample stimulus for 0.3 s, followed by another 0.7 s fixation on the white square. If fixation was successfully maintained (typically, <2.5°), the encoding phase ended with the presentation of a single drop of water.

The encoding phase was followed by a blank interphase delay interval of 0.7–1.4 s during which no fixation was required. The response phase was initiated using a fixation dot presented at the center of the screen. One of the six items was then presented at the center for 0.3 s as a cue stimulus. After another 0.5 s delay period, five disks were presented as choices, including a blue disk in each quadrant and a green disk at the center. When the cue stimulus was the same as the sample stimulus, the animal was required to choose by touching the blue disk in the same quadrant as the sample (i.e., the match condition). Otherwise, the subject was required to choose the green disk (i.e., the nonmatch condition). If the animal made the correct choice, four to eight drops of water were provided as a reward; otherwise, an additional 4 s was added to the standard intertrial interval (1.5–3 s). The number of reward drops was increased to encourage the animal to maintain a good performance in the latter phase of a daily recording session, which was typically conducted in blocks (e.g., a minimal set of 60 trials with equal numbers of visual items presented in a match/nonmatch condition). During the trial, a large gray square (48° on each side) was presented at the center of the display, as a background. After the end of the trial, all stimuli disappeared, and the entire screen displayed a light red color during the intertrial interval. The start of a new trial was indicated by the reappearance of a large gray square on the display, at which point the monkey could pull the lever, triggering the appearance of a white fixation dot. In the match condition, sample stimuli were pseudorandomly chosen from six well learned visual items, and each item was presented pseudorandomly within four quadrants, resulting in 24 (6 × 4) configuration patterns. In the nonmatch condition, the location of the sample stimulus was randomly chosen from the four quadrants, and the cue stimulus was randomly chosen from the remaining five items that differed from the sample. The match and nonmatch conditions were randomly presented at a ratio of 4:1, resulting in 30 (24 + 6) configuration patterns. The same six stimuli were used during all recording sessions.

### Electrophysiological recordings and data preprocessing

#### Experiment 1

We used conventional techniques to record single-neuron activity from the central part of the orbitofrontal cortex (cOFC; area 13 M). A tungsten microelectrode (1–3 MΩ; FHC) was used to record single-neuron activity. The electrophysiological signals were amplified, bandpass filtered (50–3000 Hz), and monitored using a recording system (model RZ5D, Tucker-Davis Technologies). Single-neuron activity was isolated manually based on the online spike waveforms. The activity of all single neurons was sampled when the activity of an isolated neuron demonstrated a good signal-to-noise ratio (>2.5). The signal-to-noise ratio was calculated online as the ratio of the spike amplitude and the range of the baseline voltage on the oscilloscope. Blinding of recorded neurons was not performed. The sample sizes required to detect the effect sizes (the numbers of recorded neurons, recorded trials in a single neuron, and monkeys) were estimated based on previous studies (Yamada et al., 2013; Chen and Stuphorn, 2015; Yamada et al., 2018). Neural activity was recorded during the 100–120 trials of the single-cue task. Neural activity was not recorded during the choice trials. We recorded the cOFC of a single right-side hemisphere in each of the two monkeys in the experiment, with a total of 190 cOFC neurons (98 SUN and 92 FU). In Exp. 1, only a single-neuron recording was performed online.

#### Experiment 2

To record single-unit activity, we used a 16-channel vector array microprobe (model V1 X 16-Edge, NeuroNexus), a 16-channel U-Probe (Plexon), a tungsten tetrode probe (Thomas RECORDING), or a single-wire tungsten microelectrode (Alpha Omega). The electrophysiological signals were amplified, bandpass filtered (200–6000 Hz), and monitored. Single-neuron activity was isolated based on the spike waveforms online or offline. For both clustering and offline sorting, the activity of all single neurons was sampled when the activity of an isolated neuron demonstrated a good signal-to-noise ratio (>2.5). We visually checked the signal-to-noise ratio by calculating the range of background noise against the spike amplitude, monitored online using the OmniPlex Neural Data Acquisition System or offline using the Offline Sorter software (Plexon). Blinding of recorded neurons was not performed. The sample sizes required to detect the effect sizes (the numbers of recorded neurons, recorded trials in a single neuron, and monkeys) were estimated based on previous studies (Naya et al., 2003; Naya and Suzuki, 2011). Neural activity was recorded during the 60–240 trials of the item-location-retention (ILR) task. We recorded 590 HPC neurons, among which the recording sites appeared to cover all its subdivisions (i.e., the dentate gyrus, CA3, CA1, and subicular complex).

### Statistical analysis

For the statistical analyses, we used the statistical software packages R (Exp. 1) and MATLAB (Exp. 2). All statistical tests for the neural analyses were two tailed.

### Behavioral analysis

No new behavioral results were included, but the procedure for the behavioral analysis are as follows.

Regarding Exp. 1, we previously reported that monkey behavior depends on expected values, defined as the probability time magnitude (Yamada et al., 2021). We described the analysis steps to check whether the behavior of the monkey reflected the task parameters, such as reward probability and magnitude. Importantly, we showed that the choice behavior of the monkey reflected the expected values of the rewards (i.e., probability multiplied by magnitude). For this purpose, the percentage of choosing the right-side option was analyzed in the pooled data using a general linear model with the following binomial distribution:

$$P{\text{chooses}}_{R}=\text{ 1 }/\;\left(\text{1 }+\;e^{-z}\right),$$

where the relationship between *P*chooses*_R_* and *Z* was given by the logistic function in each of the following three models: number of pie segments (M1), probability and magnitude (M2), and expected values (M3), as follows:

$$\text{M1}:Z=b_{0}+b_{\text{1}}N{\text{pie}}_{L}+b_{\text{2}}N{\text{pie}}_{R},$$

where *b*_0_ is the intercept and *N*pie*_L_* and *N*pie*_R_* are the number of pie segments contained in the left and right pie chart stimuli, respectively. Values of *b*_0_ to *b*_2_ were free parameters and were estimated by maximizing the log likelihood, as follows:

$$\text{M2}:Z=b_{0}+b_{\text{1}}P_{L}+b_{\text{2}}P_{R}+b_{\text{3}}M_{L}+b_{\text{4}}M_{R},$$

where *b*_0_ is the intercept; *P_L_* and *P_R_* are the probability of rewards for left and right pie chart stimuli, respectively, and *M_L_* and *M_R_* are the magnitude of rewards for left and right pie chart stimuli, respectively. Values of *b*_0_ to *b*_4_ were free parameters and estimated by maximizing the log likelihood, as follows:

$$\text{M3}:Z=b_{0}+b_{\text{1}}{\text{EV}}_{L}+b_{\text{2}}{\text{EV}}_{R},$$

where *b*_0_ is the intercept and EV*_L_* and EV*_R_* are the expected values of rewards as probability multiplied by magnitude for left and right pie chart stimuli, respectively. Values of *b*_0_ to *b*_2_ were free parameters and estimated by maximizing the log likelihood. We identified the best model to describe the behavior of the monkeys by comparing their goodness-of-fit values based on Akaike information criterion and Bayesian information criterion (Burnham and Anderson, 2004).

Regarding Exp. 2, we previously reported that two monkeys learned to retain item and location information of the sample stimulus (Chen and Naya, 2020). Here, we described the analysis steps to check whether the monkey used both item and location information to perform the task.

To examine the above point, we compared the actual correct rates of animals during the recording to the random correct rates (χ^2^ test). The ILR response phase has five options, resulting in a 20% random correct rate. If the animal uses a wrong strategy, such as only retaining the location information of the sample stimulus and ignoring the item information, the correct rate for the match condition would be 100% and the correct rate for the nonmatch condition would be 0. Based on the above considerations, we examined the actual correct rates of the two animals in the match and nonmatch conditions, respectively. In general, the average correct rates of both animals in the match and nonmatch conditions were well above the chance level after training.

### Neural analysis

Peristimulus time histograms were constructed for each single-neuron activity, aligned at the onset of the visual stimulus. Average activity curves were smoothed for visual inspection using a Gaussian kernel (Exp. 1, σ = 50 ms; Exp. 2, σ = 20 ms), while the Gaussian kernel was not used for the statistical tests.

To achieve comparisons between the two different datasets that were as fair as possible, we used the same criteria to analyze the neural activity. For the neural analyses, we used the following four criteria: (1) same analysis window size; (2) visual response within a short time 0.6 s; (3) neural modulations detected at the same significance level (*p* < 0.05); and (4) by using a general linear model (linear regression, Exp. 1.; ANOVA, Exp. 2). The details of these analysis procedures are shown below for the rate-coding and dynamic models.

### Rate-coding model: conventional analyses to detect neural modulations in each neuron

We analyzed neural activity for the 1 s time period (0–1 s after cue onset, Exp. 1) and 0.92 s time period (0.08–1 s after sample onset, Exp. 2). These activities were used for the conventional analyses described below. No Gaussian kernel was used.

#### Experiment 1

Neural discharge rates (*F*) were fitted using a linear combination of the following parameters:

(1) $$F\;=\;b_{0}+\;b_{p}\text{Probability}+\;b_{m}\text{Magnitude,}$$

where Probability (P) and Magnitude (M) denote the probability and magnitude of the rewards indicated by the pie chart segments, respectively. *b*_0_ is the intercept, and if *b_p_* and *b_m_* were not 0 at *p *<* *0.05, the discharge rates were regarded as significantly modulated by that variable. The regression coefficients for probability and magnitude were plotted as a time series for visual inspection. These results have been reported previously (Yamada et al., 2021).

Based on linear regression, activity modulation patterns were categorized into the following several types: Probability type with a significant *b*_p_ and without a significant *b*_m_; Magnitude type without a significant *b*_p_ and with a significant *b*_m_; Both type with significant *b*_p_ and *b*_m_.

#### Experiment 2

For neural responses during the encoding phase after the sample presentation, we evaluated the effects of “item” and “location” for each neuron using a two-way ANOVA (*p *<* *0.05 for each). We analyzed neurons that were tested in at least 60 trials (10 trials per stimulus and 15 per location). On average, we conducted 100 trials per neuron (*n* = 590). The results of the ANOVA were previously reported (Chen and Naya, 2020). The statistical significance level was set at *p *<* *0.05 in Exp. 2, same as that in Exp. 1 (*p *<* *0.05). The regression table was extracted and plotted as a time series for visual inspection.

Based on the ANOVA, activity modulation patterns were categorized into several types: Item type, with a significant main effect of the item and without Location effect; Location type, with a significant main effect of the location and without item effect; and Both type, with significant effects of Item and Location or with a significant interaction.

### Dynamic model: population dynamics for representing neural modulations

We analyzed neural activity during a 0.6 s time period from cue onset (Exp. 1) and sample onset (Exp. 2). To obtain a time series of neural firing rates within this period, we estimated the firing rates of each neuron for each 0.02 s time bin (without overlap) during the 0.6 s period. No Gaussian kernel was used. For this dynamic analysis, the standard statistical software R was used to apply (1) a regression analysis or ANOVA and (2) PCA. No other mathematical applications were used.

#### Regression subspace

We used a general linear model to determine the probability and magnitude of rewards (Exp. 1) and item and location (Exp. 2) affecting the activity of each neuron in the neural population. Each neural population was based on all recorded neurons in each brain region.

In Exp. 1, we first set probability and magnitude to 0.1 and 1.0, and 0.1–1.0 ml, respectively. We then described the average firing rates of neuron *i* at time *t* as a linear combination of the probability and magnitude in each neural population, as follows:

(2) $$F_{\left(i,t,k\right)}=\;b_{0(i,t)}+\;b_{1(i,t)}{\text{Probability}}_{\left(k\right)}+\;b_{2(i,t)}{\text{Magnitude}}_{\left(k\right)},$$

where *F*_(_*_i,t,k_*_)_ denotes the average firing rate of neuron *i* at time *t* on trial *k*, Probability_(_*_k_*_)_ denotes the probability of the reward cued to the monkey in trial *k,* and Magnitude_(_*_k_*_)_ is the magnitude of the reward cued to the monkey in trial *k*. The regression coefficients *b*_0(_*_i_*_,_*_t_*_)_ to *b*_2(_*_i_*_,_*_t_*_)_ describe the degree to which the firing rates of neuron *i* depend on the mean firing rates (hence, firing rates independent of task parameters), probability of rewards, and magnitude of rewards respectively, at a given time *t* during the trials. To construct the regression matrix, this information was obtained in the regression table for linear regression, which represents the coefficient for the probability and magnitude.

In Exp. 2, we first set six items and four locations as categorical parameters. We then described the average firing rates of neuron *i* at time *t* as a linear combination of item and location in each neural population, as follows:

(3) $$F_{\left(i,t,k\right)}=\;b_{0(i,t)}+\;b_{1(i,t)}{\text{Item}}_{\left(k\right)}+\;b_{2(i,t)}{\text{Location}}_{\left(k\right)},$$

where *F*_(_*_i_*_,_*_t_*_,_*_k_*_)_ denotes the average firing rate of neuron *i* at time *t* in trial *k*, Item_(_*_k_*_)_ denotes the type of item cued to the monkey in trial *k*, and Location_(_*_k_*_)_ is the type of location cued to the monkey in trial *k*. Each of the regression coefficients *b*_0(_*_i_*_,_*_t_*_)_, *b*_1(_*_i_*_,_*_t_*_)_, and *b*_2(_*_i_*_,_*_t_*_)_ describe the degree to which the firing rates of neuron *i* depend on the mean firing rates (*b*_0_, firing rates independent of task parameters), the degree of the firing rate in each item relative to the mean firing rates (*b*_1_, composed of six coefficients for corresponding items), and the degree of firing in each location relative to the mean firing rates respectively (*b*_2_, composed of four coefficients for corresponding locations), at a given time *t* during the trials. The interaction term was not included in the model because of the linear assumption in the state-space analysis. To construct the regression matrix, this information was obtained in the regression table for ANOVA, which represents the averaged activity differences in both item and location from their mean.

In Exp. 1 and Exp. 2, we used the regression coefficients (regression table in the case of ANOVA) described in Equations 2 and 3 to identify how the dimensions of the neural population signals were composed of information related to probability and magnitude (Exp. 1) and to item and location (Exp. 2). This step constructed an encoding model in the subspace, where the regression coefficients could be represented by the temporal structure of the neural modulation of two continuous parameters (Exp. 1) or two categorical parameters (Exp. 2) at the population level. In this study, our orthogonalized task design allowed us to reliably project neural firing rates into a regression subspace. In Exp. 1., two continuous parameters for probability and magnitude (10 × 10) were orthogonalized in the data. In Exp. 2., two categorical parameters for item and location (6 × 4) were orthogonalized in the data. Our procedures were analogous to the state-space analysis performed by Mante et al. (2013), in which regression coefficients were used to provide an axis (or dimension) of the parameters of interest in a multidimensional state space obtained through PCA. However, our analysis was different from that of Mante et al. (2013), in terms of describing the temporal structures of neural modulations (i.e., neural dynamics in the regression subspace).

#### Principal component analysis

We used PCA to identify the dimensions of the neural modulation signal in the orthogonal spaces composed of the probability and magnitude of rewards in Exp. 1 and items and locations in Exp. 2 for each neural population. In each neural population, we first prepared a two-dimensional data matrix *X* of size *N*_(_*_n_*_)_ × *M*_(_*_C_*_×_*_T_*_)_; regression coefficient vectors *b*_1(_*_i_*_,_*_t_*_)_ and *b*_2(_*_i_*_,_*_t_*_)_ in Equations 2 and 3, whose rows correspond to the total number of neurons (*n*) in each neural population, while the columns correspond to *C*, the total number of conditions (i.e., probability and magnitude in Exp. 1, six items and four locations in Exp. 2). *T* is the total number of analysis windows; hence, time (i.e., 30 bins:0.6 s divided by the window size bin 0.02 s). A series of eigenvectors of the covariance matrix of *X* was obtained by applying PCA one time to the data matrix *X* in each neural population. We used the prcomp () function in the R software. The two-dimensional regression matrix X was not normalized. The principal components (PCs) of this data matrix consisted of vectors *v*_(_*_a_*_)_ of length *N*_(_*_n_*_)_ and the total number of recorded neurons, if *M*_(_*_C_*_×_ *_T_*_)_ is greater than *N*_(_*_n_*_)_; otherwise, the length was *M*_(_*_C_*_×_ *_T_*_)_. The PCs were indexed from those explaining most of the variance to the least. We did not include the intercept term *b*_0(_*_i_*_,_*_t_*_)_ to focus on neural modulation using the parameters of interest.

#### Eigenvectors

When we applied PCA to the data matrix *X*, we obtained the eigenvectors and eigenvalues of the covariance matrix of *X*. Each eigenvector had a corresponding eigenvalue. In our analysis, the eigenvectors at time *t* represented vectors in the space of probability and magnitude in Exp. 1 and six items and four locations in Exp. 2. The eigenvalues at time *t* for probability and magnitude in Exp. 1 and items and locations in Exp. 2, respectively, indicate the extent of variance in the data of that vector. Therefore, the first PC is the eigenvector with the highest eigenvalue over time. We analyzed the eigenvectors for the first three PCs (PC1 to PC3). We applied PCA one time to each neural population; thus, the total variance contained in the data differed among the neural populations.

#### Analysis of eigenvectors

We evaluated the characteristics of eigenvectors for PC1 to PC3 in each neural population in terms of vector angle, size, and deviance in the space of probability and magnitude in Exp. 1, and in terms of items and locations in Exp. 2, respectively. The angle is the vector angle from the horizontal axis of 0°, from −180° to 180° against the main PCs. The size is the length of the eigenvector, and the deviance is the difference between vectors. We estimated deviance from the mean vector for each neural population. These three characteristics of the eigenvectors were compared in each neural population at *p *<* *0.05, using the Kruskal–Wallis and Wilcoxon rank-sum tests. The vector during the first 0.1 s was extracted from these analyses.

#### Shuffle control for PCA

We performed three shuffle controls to examine the significance of the population structures described by PCA. A two-dimensional data matrix *X* was randomized by shuffling in three ways. In shuffle condition 1, matrix *X* was shuffled by permutating the allocation of neuron *i* at each time *t*. This shuffle provided a data matrix *X* of size *N*_(_*_n_*_)_ × *M*_(_*_C_*_×_ *_T_*_)_, eliminating the temporal structure of neural modulation by condition *C* in each neuron but retaining neural modulations at time *t* at the population level. In shuffle condition 2, matrix *X* was shuffled by permutating the allocation of time *t* in each neuron *i*. This shuffle provided a data matrix *X* of size *N*_(*n*)_ × *M*_(_*_C_*_×_ *_T_*_)_, eliminating the neural modulation structure under condition *C* maintained in each neuron, but retaining neural modulation in each neuron at the population level. In shuffle condition 3, matrix *X* was shuffled by permutating the allocation of both time *t* and neuron *i*. In these three shuffle control conditions, matrix *X* was estimated 1000 times. PCA performance was evaluated by constructing the distributions of the explained variances for PC1 to PC12. The statistical significance of the variances explained by PC1 and PC3 was estimated based on the 95th percentile of the reconstructed distributions of the explained variance or bootstrap SEs (i.e., the SD of the reconstructed distribution).

#### Preference ordering

In Exp. 2, each neuron had a preferred item and location, as in the conventional rate-coding model analysis. We defined the preferred item and location in each neuron to construct matrix *X*. We constructed *X* with and without rank order. Items 1–6 were rank ordered from most to least preferred, defined as the mean firing rate during a whole analysis time window from 0.08 to 1 s. Thus, Item_(_*_k_*_)_ was the rank-ordered item cued to the monkey on trial *k.* Similar to the definition of Item, Location_(_*_k_*_)_ was the rank-ordered location cued to the monkey during trial *k*. This preference ordering did not change over time *t* for each neuron *i*.

#### Optimal stimulus response analysis in the dynamic model

As typical single-neuron analyses assess optimal and nonoptimal conditions (i.e., the best and worst), we linked the optimal response analysis to the dynamic analysis of the neuronal population as follows.

We used only two columns in each time bin, the most and least preferred, for each condition *C*, item, and location. Thus, matrix *X* was *X* of size *N*_(590)_ × *M*_(4 × 30)_. This corresponded to the conventional analysis used in the rate-coding model, which compares neuronal responses between the most and least preferred conditions, but for the evaluation of neural modulation dynamics in these two conditions. We evaluated the percentage explained by the model between the original and restricted matrices in the HPC for categorical task parameters.

### Data availability

All data and analysis codes in this study are available from the corresponding authors.

## Results

We proposed and tested a dynamic analysis method that could be applied to existing datasets using a typical factorial design for conventional single-neuron recordings (Fig. 1; Exp.1; Chen and Naya, 2020), which is in line with the compatible analysis we have previously developed for the continuous task parameters (Exp. 2; Yamada et al., 2021).

### Tasks, behavior of monkeys, and datasets

**Figure 2.**
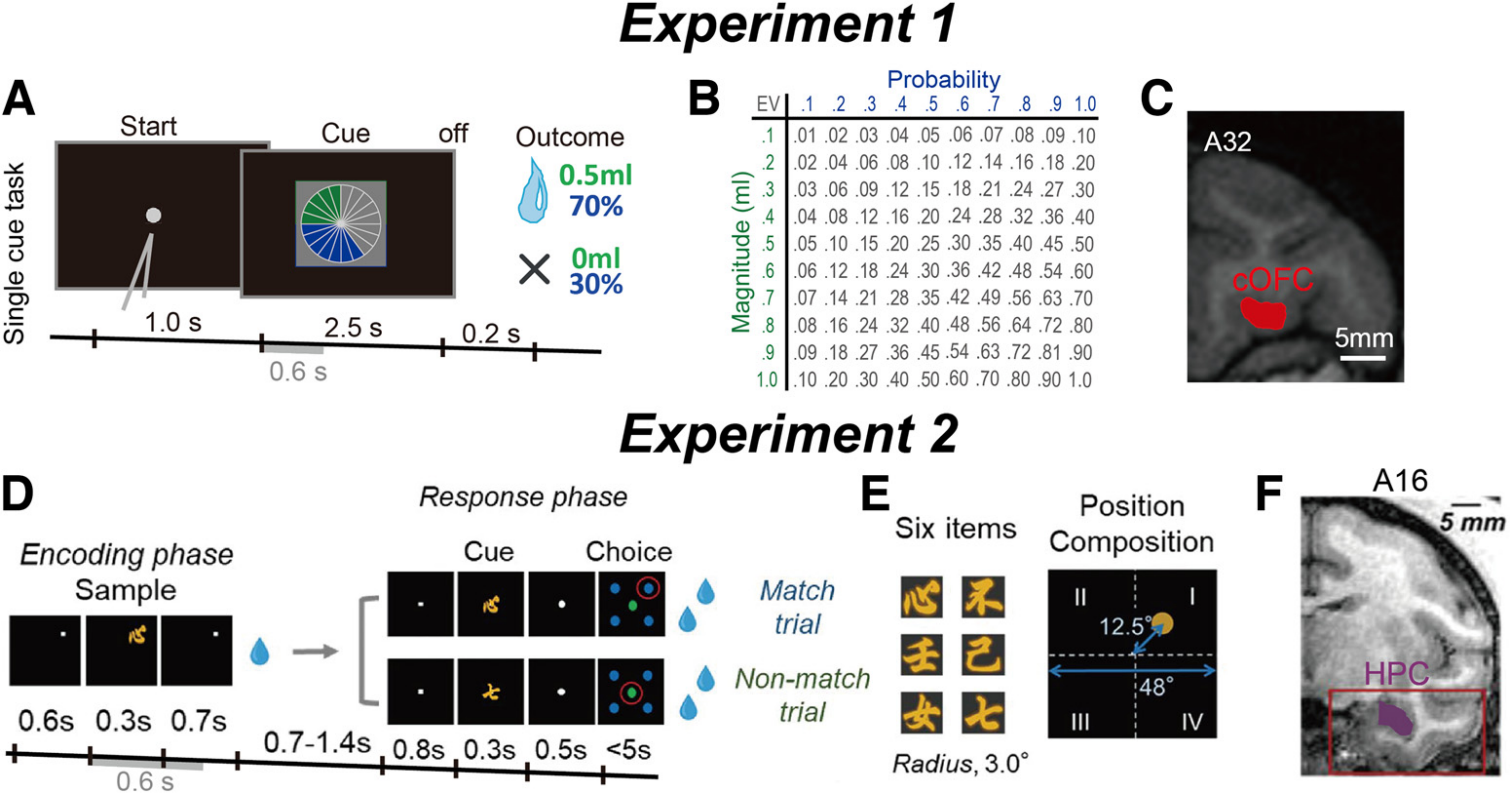
Behavioral task and recording location of neurons. ***A***, The sequence of events during the single-cue task in Exp. 1. A single visual pie chart containing green and blue pie segments was presented to the monkeys. Neural activity was analyzed during the initial 0.6 s after cue onset, that is, for the same duration as in Exp. 2. ***B***, Payoff matrix: each of the magnitudes was fully crossed with each probability resulting in a pool of 100 lotteries. ***C***, Illustration of neural recording areas based on coronal magnetic resonance (MR) images for the cOFC (13 M, medial part of area 13) at the A31–A34 anterior–posterior level. ***D***, The sequence of events during the ILR task in Exp. 2. The cue stimulus during the response phase was the same as the sample stimulus during the encoding phase in the match trial, whereas the two stimuli differed in the nonmatch trial. Neural activity was analyzed during the initial 0.6 s after sample onset, that is, for the same duration as in Exp. 1. ***E***, Six visual item stimuli and spatial composition for the sample stimulus. ***F***, Coronal MR images from monkey A for the HPC population showing the recording area at A16–A10.5, depicted in purple within the red box. ***A*** was published previously in the study by Yamada et al. (2021). ***D–F*** was published previously in the study by Chen and Naya (2020).

During the cued lottery task in Exp. 1 using two continuous task parameters (Fig. 2*A*), the monkeys estimated the expected value of the lottery, defined as a multiplicative combination of probability and magnitude (Fig. 2*B*; probability, 0.1–1.0 in 0.1 increments; magnitude, 0.1–1.0 ml in 0.1 ml increments) and chose the option with higher expected values when two options were presented (Yamada et al., 2021). This choice behavior was observed and analyzed separately from the neural recordings (see Materials and Methods). We analyzed neuronal activity recorded from the cOFC (Fig. 2*C*) in the nonchoice condition, where a single lottery cue and its outcome were provided to the monkeys (Fig. 2*A*). For neural analyses, information on the probability and magnitude of rewards provided by the cue stimulus was used for continuous task parameters.

In Exp. 2 using two categorical task parameters, the monkeys retained the visual items and their locations during the encoding phase, after which they indicated whether the sample item matched the cued items by choosing the memorized location (Match trial) or not by choosing the central green disk (Nonmatch trial; Fig. 2*D*; see Materials and Methods). Six visual items and four locations were used (Fig. 2*E*). After completing training, average correct rates were well above the chance level (Chen and Naya, 2020). We analyzed neural activity recorded from the HPC (Fig. 2*F*) when the sample stimulus was presented to the monkeys during the encoding phase. For neural analyses, information on the items provided by the sample stimulus and the locations were used as categorical task parameters, whereas the location information had already been provided to the monkeys before the sample appearance. Details of the behavioral training, learning progress, and behavioral performance of the animals in the cued lottery (Exp. 1; Yamada et al., 2021) and in the ILR tasks (Exp. 2; Chen and Naya, 2020) have been reported previously.

We constructed two different sets of pseudopopulations of neurons using the single-neuron activity of the cOFC (Fig. 2*C*, 190 neurons) in a 0.6 s time period with respect to the lottery cue onset in the single-cue task (Fig. 2*A*, gray bar) and HPC (Fig. 2*F*, 590 neurons) in a 0.6 s time period with respect to the sample onset in the ILR task (Fig. 2*D*, gray bar). We denote that the HPC population data in Exp. 2 were analyzed and previously reported using a rate-coding model (Chen and Naya, 2020), but this has not been analyzed using a dynamic model. The cOFC population data from Exp. 1 was analyzed and reported previously using both rate-coding and dynamic models (Yamada et al., 2021), and we repeated the same analysis with a shorter analysis time period (2.7 s was used previously) for comparisons between the neural modulation dynamics in Exp.1 and Exp. 2.

### Neural recordings and data preprocessing

To achieve the fairest possible comparisons between the two distinctive datasets, we adhered to the following criteria for neural recordings and analyses. First, for the recordings and preprocessing of the neural data, datasets in Exp. 1 and Exp. 2 were individually prepared, because each experimenter has their own criteria for single-neuron activity. To ensure that the dataset differences were reliable, we provided the detailed procedure for recording single-neuron activity in the Materials and Methods, including bandpass filtering, signal-to-noise ratio, and online or offline sorting. Second, for the neural analyses we used a similar size of analysis window and statistical procedure because analysis window size is critical for detecting neural modulations in both conventional and dynamic analyses. A window of ∼1.0 s was used for the conventional rate-coding analyses with a fixed time window (Exp. 1, 0–1.0 s; Exp. 2, 0.08–1.0 s). A 0.02 s time bin was used for the analysis of time-dependent neural modulation 0.6 s after the visual stimulus appeared in both experiments. Third, for statistical procedures, we used the general linear model at *p* < 0.05 (linear regression for the continuous task parameter in Exp. 1 and ANOVA for the categorical task parameter in Exp. 2). Based on these criteria, we described the neural response and its dynamics in fine time resolution (0.02 s) during a short time period (0.6 s) after the appearance of visual stimulus, during which continuous and categorical task parameters were processed by the neurons.

### Conventional analyses for detecting neural modulations by task parameters

**Figure 3.**
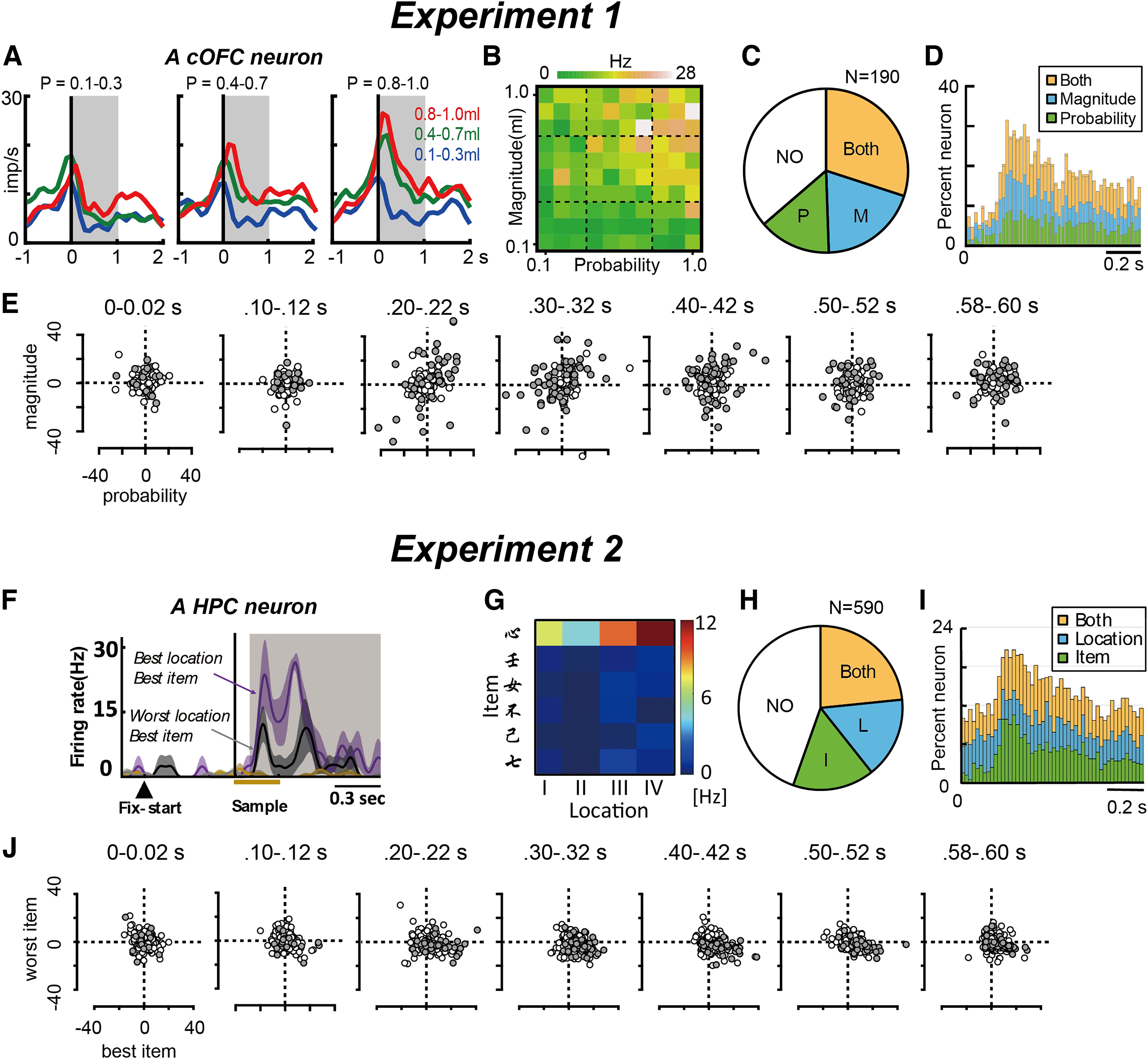
Example activity of neurons during the single-cue and ILR tasks. ***A***, An example activity histogram of a cOFC neuron modulated by the probability and magnitude of rewards during the single-cue task. Activity aligned with cue onset is represented for three different levels of probability (P, 0.1–0.3, 0.4–0.7, 0.8–1.0) and magnitude (M, 0.1–0.3 ml, 0.4–0.7 ml, 0.8–1.0 ml) of rewards. Gray hatched areas indicate the 1 s time window used to estimate the neural firing rates shown in ***B***. Histograms smoothed using a Gaussian kernel (σ = 50 ms). ***B***, An activity plot of the cOFC neuron during the 1 s time window shown in ***A*** against the probability and magnitude of rewards. ***C***, The percentage of neural modulation types detected in 1 s time window shown in ***A***: the P, M, Both, and NO. ***D***, Percentages of neural modulation type detected in the 0.02 s time bins during the 1.0 s after cue onset. Calibration: 0.2 s. ***E***, Regression coefficient plots for the probability and magnitude of rewards estimated for all cOFC neurons in Exp. 1. Regression coefficients in the 0.02 s time bin shown every 0.1 s during the 0.6 s after cue onset (0–0.02 s, 0.10–0.12 s, 0.20–0.22 s, 0.30–0.32 s, 0.40–0.42 s, 0.50–0.52 s, and 0.58–0.60 s). Filled gray indicates significant regression coefficient for either Probability or Magnitude at *p* < 0.05. ***F***, An example of an HPC neuron showing sample-triggered sample–location signals and item signals. A 0.08–1.0 s time window after sample onset was used to estimate the neural firing rates shown in ***G***. Histograms are smoothed using a Gaussian kernel (σ = 20 ms). ***G***, An activity plot of the HPC neuron during the time window shown in ***F*** against item and location. ***H***, The percentage of neural modulation types detected in the 0.08–1.0 s window shown in ***F***; Item, Location, Both, and NO. ***I***, Percentages of neural modulation types detected in the 0.02 s time bins during the 1.0 s after sample onset. ***J***, Regression coefficient plots for the best and worst items estimated for all HPC neurons in Exp. 2. Filled gray indicates significant regression coefficient for item at *p* < 0.05 using ANOVA without interaction term. The location modulation was not shown because we showed changes of neural modulation by the sample stimulus, whereas the location had already been provided to the monkeys. ***A***, ***B***, and ***D*** were published previously in the study by Yamada et al. (2021).

We first applied common conventional analyses, such as the general linear model typically used in rate-coding models (linear regression in Exp. 1 and ANOVA in Exp. 2; see Materials and Methods). In Exp. 1, we examined how the probability and magnitude of rewards were encoded and integrated by the activity of OFC neurons immediately after cue presentation. Linear regression analysis revealed that cOFC neurons encode both probability and magnitude to some extent after cue onset, as shown in an example neuron (Fig. 3*A*,*B*; *n* = 119 trials; coefficient/intercept: −0.74; *t* = −0.72, *p *=* *0.47; probability: 8.55, *t* = 6.91, *p *<* *0.001; magnitude: 11.1, *t* = 8.95, *p *<* *0.001). In the cOFC populations, approximately half of the neurons were modulated by the probability and magnitude of rewards during the 1 s time window (0–1 s after cue onset; probability: 44%, 84 of 190; magnitude: 49%, 94 of 190). Modulations for either or both probability and magnitude were found (Fig. 3*C*; Both: 30%, 57 of 190; P: 14.2%, 27 of 190; M: 19.5%, 37 of 190; Nonmodulated (NO): 36.3%, 69 of 190). The analysis with 0.02 s time bins showed that the percentage of neurons modulated by these two parameters increased, reached a maximum percentage at ∼0.25 s, and then gradually decreased during the 1.0 s after the onset of the lottery cue (Fig. 3*D*).

In Exp. 2, we analyzed neural modulations using the following two categorical task parameters: the item and location after the sample presentation. The analyses were aimed at examining how the item information was encoded and integrated with the location information by the neurons immediately after the sample presentation. ANOVA revealed that HPC neurons encoded both item and location information to some extent, as shown in an example neuron (Fig. 3*F*,*G*; two-way ANOVA, *n* = 120 trials; item: *F*_(5,96)_ = 52.1, *p *<* *0.0001; location: *F*_(3,96)_ = 4.4, *p *=* *0.006). In the HPC population, neurons were modulated by these two factors (0.08–1 s after sample onset; Item, 39%, 232 of 590; Location, 39%, 233 of 590). Modulations for either or both factors were found (Fig. 3*H*; Both: 23%, 138 of 590; Item: 16%, 95 of 590; Location: 16%, 94 of 590; NO: 45%, 263 of 590). The analysis with the 0.02 s time bins showed that the percentage of neurons modulated by these two factors increased, reached a maximum percentage at ∼0.25 s, and then gradually decreased during the 1.0 s after the onset of the sample stimulus (Fig. 3*I*). We note that the percentage of neural modulation by the presented location did not change with time because the monkeys already knew the item location before the sample presentation (Fig. 3*I*, see blue), whereas the percentage change in neural modulations was because of an increase in item information (Fig. 3*I*, see green). Thus, for both neural populations, typical changes in neural modulation were observed as a percentage increase, followed by a decrease after visual stimulus onset in the conventional analysis.

We then examined a regression coefficient that describes the extent of neural modulations in each neural population. In Exp. 1, the regression coefficients for the probability and magnitude of rewards in the case of continuous variables were visualized in the cOFC population (Fig. 3*E*). The extent of neural modulations increased between 0.2 and 0.4 s after cue onset, as seen in the larger positive or smaller negative regression coefficients for probability and magnitude. For the categorical parameters in Exp. 2, we plotted the regression coefficients represented in the ANOVA table for the best and worst items detected in each neuron because we mainly focused on the neural modulation by the visual item (Fig. 3*J*; data not shown for all visual items). The distribution of coefficients for the best items seemed to be wider at 0.2–0.5 s (see *x*-axis, especially larger positive values), compared with that just after the sample onset (0–0.02 s plots within a range of −20 to 20). Thus, for both neural populations, an increase in neural modulation was observed in the regression coefficients within a reasonable time period, compatible with the changes in the proportion of significant neural modulations. These changes in regression coefficients were used for further analyses of neural modulation dynamics.

**Figure 4.**
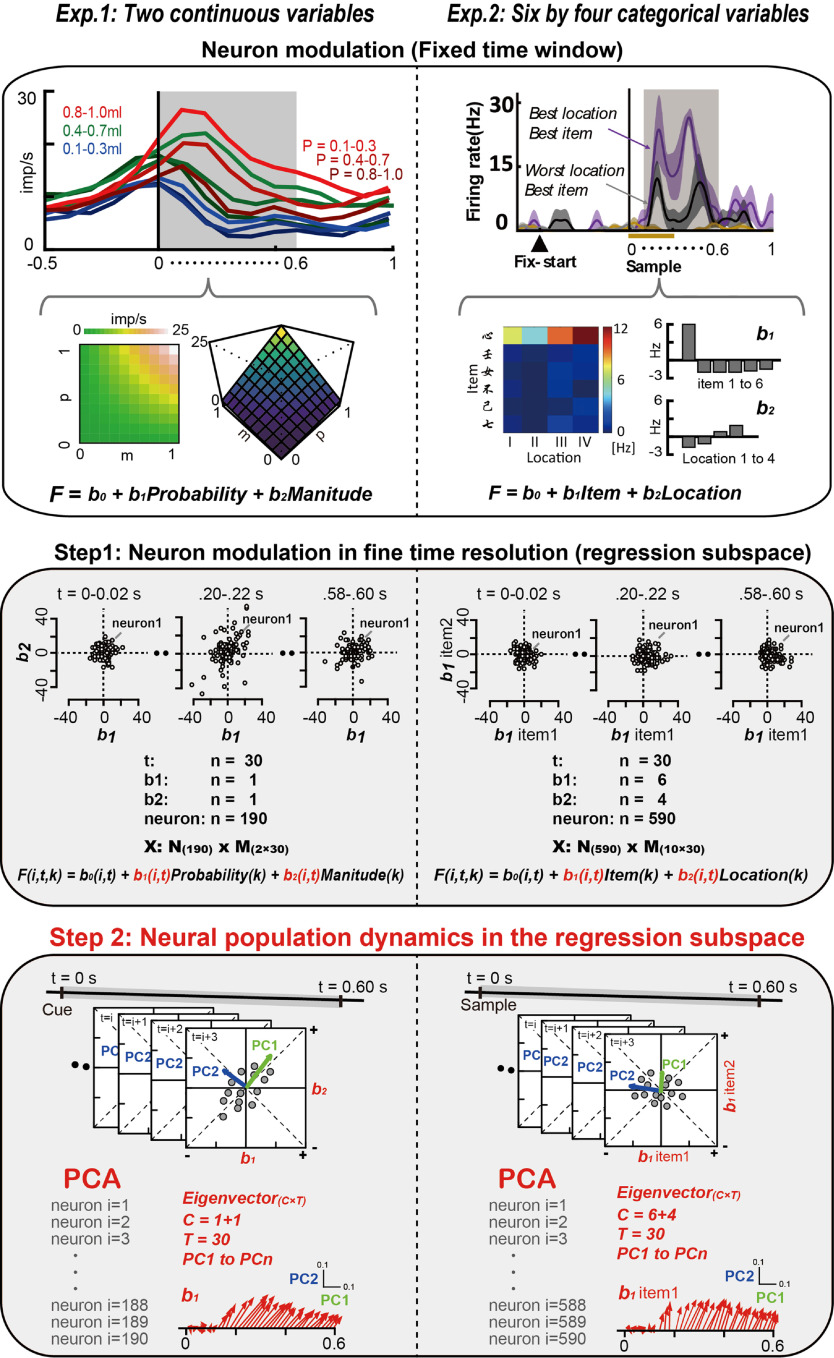
Graphic methods for the conventional rate-coding analysis and state-space analysis in the regression subspace. Conventional analysis (top and middle rows): in each single neuron, activity modulations by task variables are detected in the fixed time window (top row) using linear regression and ANOVA for continuous (left, Exp. 1) and categorical (right, Exp. 2) task parameters (Fig. 2, see for the task details), respectively. The same analyses were applied in a fine time resolution in Exp. 1 and Exp. 2 (middle row). The conventional analyses using a general linear model (linear regression and ANOVA) provide the extent of neural modulations as the coefficients in the analysis table of the statistical software. These are neural modulations in a fine time resolution observed at the level of population. *i* represents the number of neurons in each neural population (Exp. 1, 190 neurons; Exp. 2, 590 neurons). *t* represents the number of time bins (30 for both Exp. 1 and Exp. 2). In our state-space analysis as step 1, the time series of neural population activity was projected onto a regression subspace composed of probability and magnitude (left, Exp. 1) and item and location (right, Exp. 2). The middle row, therefore, represents the neural population activity in the regression subspace *X*. By applying PCA to *X*, eigenvectors for probability and magnitude were extracted and plotted after coordinate transformation against PC1 and PC2 (step 2, left, Exp. 1). Eigenvectors for item and location were plotted after coordinate transformation against PC1 and PC2 (right, Exp. 2). A series of eigenvectors was obtained by applying PCA once to the cOFC and HPC populations, respectively. The number of eigenvectors obtained by PCA was 0.6 s, divided by the analysis window size, 0.02 s, for P and M; in total, 30 eigenvectors for each (left, Exp. 1), and for the six items and four locations; in total, 30 eigenvectors for each. Extended Data Figure 4-1 represents detail of the vector analyses.

Collectively, we showed that the general linear model usually used in the rate-coding model detects neural modulations using the same continuous and categorical parameters as a standard analysis procedure. This conventional approach can provide temporal changes in the selected metrics (e.g., proportion and extent of neural modulations; Fig. 4, top row), but they cannot provide trajectory geometry at the lower dimension. We note that detailed results from these conventional analyses have been previously reported (Chen and Naya, 2020, their Figs. 2, 5; Yamada et al., 2021, their Fig. 2E,F,K,O).

10.1523/ENEURO.0016-23.2023.f4-1 Figure 4-1 Schematic depictions of eigenvector evaluations. Characteristics of the eigenvectors evaluated quantitatively. Angle, vector angle from the horizontal axis obtained from –180° to 180°; Size, eigenvector length; Deviance, difference between vectors. Download Figure 4-1, file.

Before describing the dynamic analysis, we also noted other task differences. First, information on two variables was simultaneously provided to the monkeys in Exp. 1, while the item information was provided to the monkeys following the presentation of location information in Exp. 2. Second, the monkeys simply looked at the visual cue in Exp. 1, while the monkeys were required to make choices using the item and location information later in the task in Exp. 2. Third, the monkeys made eye movements without choices in Exp. 1, while the monkeys made arm movement to choose in Exp. 2. Our further analyses provide a temporal structure of neural modulation signals, which links these rate-coding results with a dynamic system perspective.

### State-space analysis for detecting the temporal structure of neural modulation at the population level

We previously developed a variant of state-space analysis, which extracts temporal structures of neural modulations by task-related continuous parameters using standard statistical software (see Yamada et al., 2021). Here, we extend this analysis to neural modulations using categorical parameters to describe how the HPC neural population dynamically represents item and location information.

First, we used a general linear model to project a time series of each neural activity into a regression subspace composed of task parameters as continuous and categorical, as shown in the regression equations in Figure 4 (middle row, step 1; for details, see Materials and Methods). This step captures the across-trial variance caused by task-related parameters moment-by-moment at a population level, which demonstrates the extent of neural modulations by the task parameters across time. This corresponds to the estimation of the regression coefficients shown in Figure 3, *E* and *J* (for all time bins and conditions), which constructs the regression matrices detected in each neural population with a fine time resolution (Fig. 4, middle row, step 1, *X*). Second, we applied PCA one time to the time series of neural activity in the regression subspace in each neural population (Fig. 4, bottom row, step 2). This step determines the main features of the neural modulation signal across time in the predominant dimensions as trajectory geometry. These two steps identify how neural modulations by task parameters change as a time series of eigenvectors in the regression subspace.

We evaluated the properties of the extracted time series of the eigenvectors in the lower-dimensional space: the first three principal components (PC1 to PC3) in each neural population, in terms of vector angle, size, and deviance (Extended Data Fig. 4-1). The angles and sizes provide trajectory geometry that describes how neural modulation evolves after the visual presentation. Deviance indicates the stability of neural modulation between vectors. We compared two neural populations recorded during two different cognitive tasks in terms of these vector properties. We note that the typical population dynamics previously found were straight, curvy, and rotational structures (Fig. 1, right).

### Neural population dynamics representing continuous and categorical parameters

**Figure 5.**
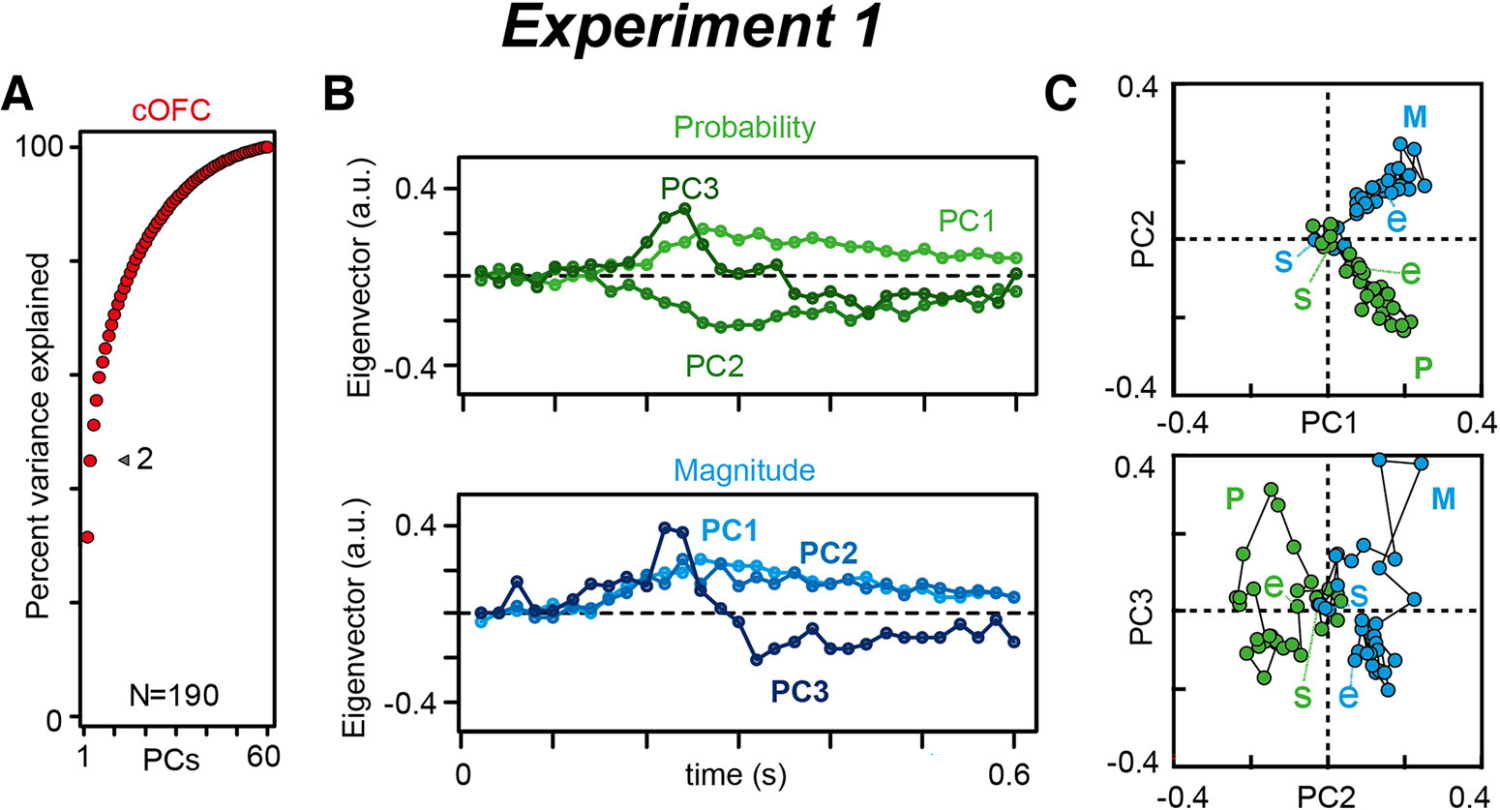
The state-space analysis provides a temporal structure of neural modulation in the cOFC. ***A***, Cumulative variance explained by PCA in the cOFC population. The arrowhead indicates the percentage of variance explained by PC1 and PC2. ***B***, Time series of eigenvectors, PC1 to PC3 in the cOFC population. ***C***, A series of eigenvectors for PC1 to PC3 are plotted against PC1 and PC2, and PC2 and PC3 dimensions in the cOFC population. Plots at the beginning and end of the series of vectors are labeled as start (s) and end (e), respectively. a.u., Arbitrary unit.

**Figure 6.**
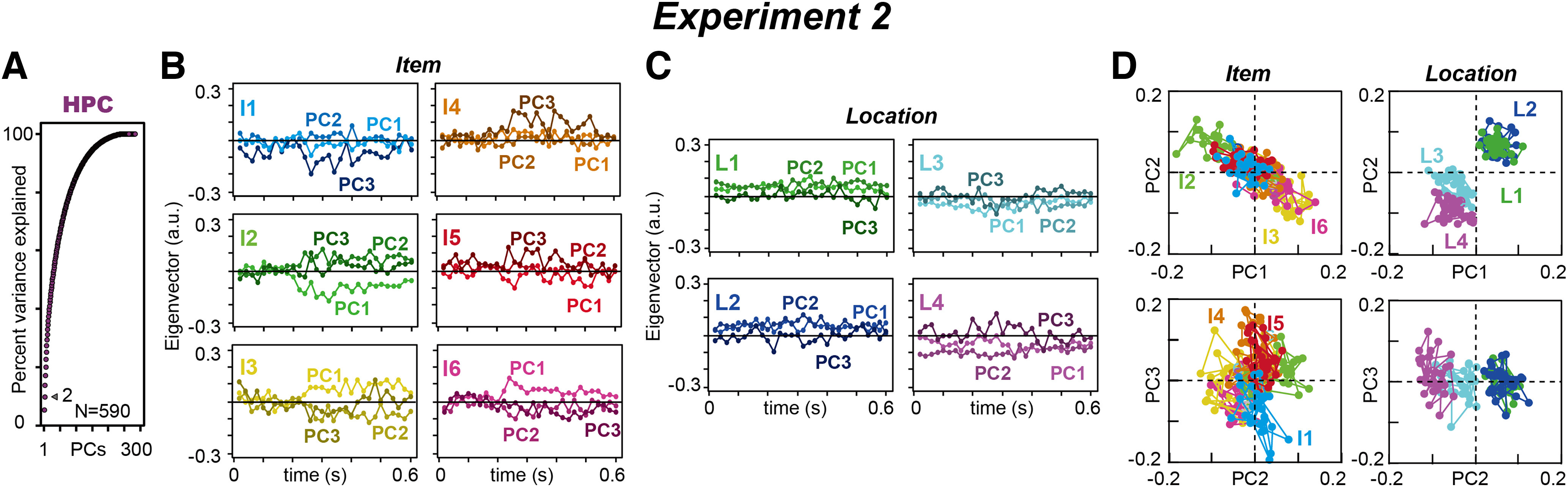
Temporal structure of neural modulation in the HPC population. ***A***, Cumulative variance explained by PCA in the HPC population. The arrowhead indicates the percentages of variances explained by PC1 and PC2. ***B***, Time series of eigenvectors for six items in the HPC population. The top three PCs are shown. ***C***, Time series of eigenvectors for four locations. ***D***, A series of eigenvectors for PC1 to PC3 are plotted against PC1 and PC2, and PC2 and PC3 dimensions in the HPC population. a.u., Arbitrary unit. Extended Data Figure 6-1 represents shuffled control results.

We first qualitatively explained how our state-space analysis describes neural population dynamics in the regression subspace in cOFC (Fig. 5) and HPC (Fig. 6) populations during the perception of visual stimuli. Using the identical analysis window size between the two experiments, we ensured that the neural population structures were comparable between the cOFC and HPC populations, with continuous and categorical parameters.

10.1523/ENEURO.0016-23.2023.f6-1 Figure 6-1 Explained variances by PCA in shuffled controls. ***A***, A boxplot of explained variances by PCA for PC1 to PC6 for the cOFC population under the three shuffled conditions (for details, see Materials and Methods). The plot is not cumulative. The boxplot was made with 1000 repeats of the shuffle in each condition. ***B***, A boxplot of explained variances by PCA for PC1 to PC12 for the HPC population. In ***A*** and ***B***, the colored circles indicate variances explained by PCA in each neural population without the shuffles. ***C***, Examples of a series of eigenvectors for PC1 to PC2, plotted in the cOFC population under the three shuffle conditions. ***D***, Examples of a series of eigenvectors for PC1 to PC2, plotted in the HPC population under the three shuffle conditions. Download Figure 6-1, file.

We first confirmed the performance of the state-space analysis, as indicated by the percentage of variance explained in the cOFC population (Fig. 5*A*). In the cOFC population, >40% of the variance was explained by PC1 and PC2 (gray arrowhead). We then characterized the entire structure of the cOFC population by plotting its eigenvectors moment-by-moment in their temporal order. As shown in Figure 5, *B* and *C*, the eigenvectors for PC1 and PC2 evolved <0.2 s after the onset of the cues in both probability and magnitude. The eigenvectors then shortened after ∼0.3 s. The angle of the eigenvectors evolved in the 45° direction in a stable manner in the PC1–PC2 plane (Fig. 5*C*, top), whereas the vectors in PC3 changed in the opposite direction, from positive to negative, over time (Fig. 5*B*,*C*, bottom). These stable structures in the top two dimensions were consistent with our previous results even when the analysis window sizes differed: a whole cue period of 2.7 s previously, but here, only the initial 0.6 s were analyzed (Yamada et al., 2021, their Fig. 7B). These straight geometric changes (i.e., stable vectors in angle) indicate that almost equal neural modulations by probability and magnitude (Fig. 3*E*) evolved across the 0.6 s time period at the neural population level. Thus, the two neural modulation ratios were kept similar.

For the HPC population modulated by the two categorical parameters, the performance of the state-space analysis was lower than that in Exp. 1 (Fig. 6*A*). The first two PCs only explained ∼10% of the variance (Fig. 6*A*, gray arrowhead). Because the percentage of modulated neurons was similar between the two neural populations (Fig. 3*C*,*H*), this would not be because of the weaker influence of task parameters in Exp. 2 on the recorded HPC population. This may be partly because of the larger regression matrix composed of 10 vectors at each time point (six items and four locations) and a larger neural population containing 590 neurons: total *X* of size *N*_(590)_ × *M*_(300)_, as in our previous study of PCA performance, where variance captured by PCA tended to decrease as the matrix size increased (Yamada et al., 2021, their Fig. 7A).

The eigenvectors in the first three PCs appeared to describe neural population dynamics in the HPC. For example, the extracted eigenvectors for each visual item evolved within a reasonable range of time with an increase and a subsequent decrease at ∼0.2–0.5 s (Fig. 6*B*), which is consistent with our findings using typical conventional analyses, ANOVA (Fig. 3*H*,*I*). In contrast, the eigenvectors for locations did not exhibit clear trends over time (Fig. 6*C*), as location information was provided to the monkeys before the sample presentations. This is also consistent with our findings using ANOVA, in which percentages modulated by locations did not change over time (Fig. 3*I*, blue). If we plotted eigenvectors in the space of the first three PCs, they consistently evolved in one direction in the PC1 and PC2 spaces (I2, I3, and I6), or PC3 spaces (I1, I4, and I5; Fig. 6*D*, left). In contrast, the eigenvectors for locations were positioned in a constant location over time (Fig. 6*D*, top right). Unambiguously, the arrangements of the eigenvectors for item and location were orthogonalized, as seen in item representations in the second and fourth quadrants and in location representations in the first and third quadrants (Fig. 6*D*, top row). The increase and subsequent decrease in vector sizes at ∼0.2–0.5 s were similar to those in the neural modulations (Fig. 3*J*). Thus, our state-space analysis in the regression subspace may capture the neural modulation dynamics in HPC populations similar to that in cOFC populations, while reflecting continuous and categorical parameters in their neural modulations.

To summarize, the main components of the neural modulation signals for categorical task parameters were evolved for item and remained stable for location, similar to the results using ANOVA. These temporal patterns of neural modulations as trajectory geometry may be able to visualize the strength of the main neural modulations and how they change over time as the eigenvectors. Next, we quantitatively examined whether the temporal patterns of geometric changes are statistically significant.

### Effect of shuffle control on PCA performance

To validate the statistical significance of these findings, we used in three ways a shuffle control procedure (for details, see Materials and Methods) that determines the number of available dimensions in the neural population. In shuffled conditions 1 and 2, information on task-related parameters was partially shuffled in the regression subspace matrix X. In shuffle condition 1, a random permutation of neuron *i* was performed at each time *t*, eliminating the temporal neural modulation structure by condition *C* across each neuron but retaining the effect of neural modulation at each time *t* at the population level. In shuffle condition 2, a random permutation of time *t* was performed for each neuron *i*, eliminating the temporal neural modulation structure by condition *C* in each neuron, but retaining the effect of neural modulation in each neuron *i* at the population level. In shuffled condition 3, random permutations of both time *t* and neuron *i* were performed. We evaluated the performance of PCAs for each condition in each experiment.

As shown in Extended Data Figure 6-1, the three shuffle control procedures reproduced different disturbances in neural populations. In shuffle conditions 1 and 3 (Extended Data Fig. 6-1*A*, left, right; see black boxplot), the explained variance decreased compared with that from the original PCA without shuffles (red circle) in the cOFC population. In shuffle condition 2, a considerable amount of variance was explained by PCA (Extended Data Fig. 6-1*A*, middle, black boxplot). These effects are consistent with those of our previous study (Yamada et al., 2021, their Fig. 5A,E,I). Since the eigenvectors were very stable across time in the cOFC population (Fig. 5*B*,*C*), the shuffle within each neuron largely did not reduce PCA performance. Indeed, some stable population structures were observed under the shuffle within each neuron (Extended Data Fig. 6-1*C*, middle) located in the first and fourth quadrants through a trial, although the trajectory geometry observed without shuffles (Fig. 5*C*, top) was impaired. In the first and third shuffle conditions, the neural population structures were completely impaired (Extended Data Fig. 6-1*C*, left, right, respectively), indicating that the shuffle across neurons strongly affected neural modulation structures in Exp. 1.

The same effects of shuffle controls were observed in the HPC population, for which categorical parameters were used (Extended Data Fig. 6-1*B*, black boxplot). A considerable amount of variance was explained by PCA in shuffled condition 2 (Extended Data Fig. 6-1*B*, middle), although the maintained population structures were unclear (Extended Data Fig. 6-1*D*, middle) compared with those in Exp. 1 (Extended Data Fig. 6-1*C*, middle). When examining the details of the decreased performance in each experiment, the performances of the first 3 and 12 PCs were better than those in the shuffled control condition 2 in Exp. 1 and Exp. 2, respectively (shuffle condition 2, *p* < 0.05). Thus, while the total number of available dimensions differed between the experiments, all three shuffles revealed the significance of the lower-dimensional neural modulation space. Note that the smaller variance explained by the top-two dimensions in Exp. 2 compared with that in Exp. 1 must be partly because of the availability of the higher dimensions in Exp. 2 (Exp. 1 explains ∼50% of variances by the top 3 PCs; Exp. 2 explains >30% by top 12 PCs).

### Activity preference and neural population dynamics

**Figure 7.**
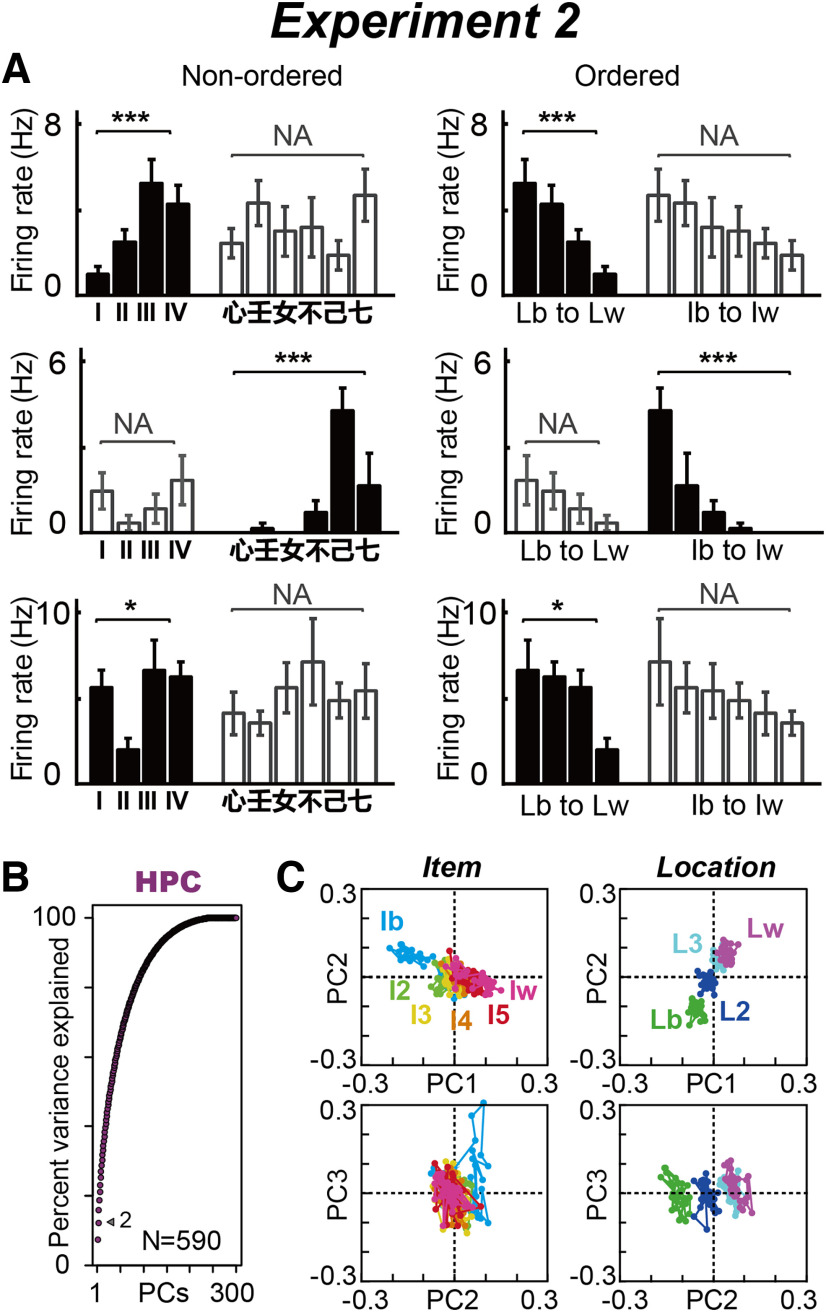
Effects of preference ordering on the HPC categorical data. ***A***, Three examples of HPC neurons for preference ordering. The activities were ordered by their preference to the items and locations (right, shown best to worst), while their activity have a preference to item or location during the 0.08–0.6 s after sample presentation. ***B***, Cumulative variance explained by PCA in the HPC population when item and location were arranged in the order of their activity preferences (see Materials and Methods). The arrowhead indicates the percentages of variances explained by PC1 and PC2. ***C***, Series of eigenvectors for PC1 to PC3 when item and location were arranged in the order of their preferences, plotted against the PC1 and PC2, and PC2 and PC3 dimensions in the HPC population. Ib and Iw indicate the best and worst items, respectively. I2 to I5 indicate the second to fifth best items. Lb and Lw indicate the best and worst locations, respectively. L2 and L3 indicate the second and third best locations, respectively. NA, No significant difference using ANOVA at *p* < 0.05. *p < 0.05, ***p < 0.001. Extended Data Figure 7-1 represents results for optimal response analysis.

In the conventional rate-coding analysis, the preferred/nonpreferred activity of neurons is accumulated to evaluate the extent of neural modulations at the population level. In Exp. 2, each neuron possesses a particular preference for item and location (Fig. 7*A*, left), and we detected the best and worst conditions in addition to all remaining conditions by ordering the activity magnitudes (Fig. 7*A*, right). In this way, we reconstructed the regression subspace in line with the conventional perspective, such as activity preference to task conditions, item and location (see Materials and Methods). This analytic approach allowed us to examine how the conventional analytic framework affects the neural population dynamics.

10.1523/ENEURO.0016-23.2023.f7-1 Figure 7-1 Optimal response analysis in the HPC population. ***A***, Cumulative variance explained by PCA in the HPC population when the best and worst conditions for item and location were used for the regression subspace. The gray dots indicate the percentage variance explained by PCA upon using the full matrix. The first 12 PCs are shown. ***B***, Time series of the eigenvectors for PC1 to PC3 when the best and worst items and locations were used. Ib and Iw indicate the best and worst items, respectively. Lb and Lw indicate the best and worst locations, respectively. s and e indicate the start and end of the time series of vectors, respectively. ***C***, A boxplot of explained variances by PCA for PC1 to PC12 under the three shuffled conditions (for details, see Materials and Methods). The plot is not cumulative. The boxplot was made with 1000 repeats of the shuffle in each condition. The colored circles indicate the variances explained by PCA in the HPC population without the shuffles. Download Figure 7-1, file.

We analyzed the most to least preferred conditions for six items and four locations in each neuron, in which item and location were remapped to the most to least preferred activity in each condition of item and location, individually. The neural preference was defined using whole analyzed activity in the 0.08–0.6 s analysis window in each neuron. The regression subspace comprised the same size, total *X* of size *N*_(590)_ × *M*_(300)_; however, condition *C* was changed to the most to least preferred items and most to least preferred locations.

The percentage variance explained by the model for PC1 and PC2 was almost the same in the preference-ordering analysis (Fig. 7*B*, 12%) compared with the original analysis (Fig. 6*A*, 10%). The composition of the eigenvectors for items was also similar between the analyses in the PC1 and PC2 dimensions (Fig. 7*C*, top left), located in the second and fourth quadrants from the most preferred (Ib, best item) to least preferred (Iw, worst item). However, the eigenvectors for items were clearly different in the PC3 dimension, as observed for the most preferred item (Ib; Fig. 7*C*, left bottom). The composition of eigenvectors for locations was not clearly changed by the preference ordering (Figs. 6*D*, right, 7*C*, right). These results suggest that preference ordering may affect eigenvector compositions at higher dimensions equal to or greater than PC3, and the entire compositions of eigenvectors seemed maintained at the lower dimension.

In summary, our analysis can extract temporal structures of item and location modulations with the preference ordering in the lower-dimensional subspace, describing how preferred and nonpreferred neural modulation change over time. The predominant temporal structures maintained with and without preference ordering (compare Figs. 7*C*, top row, 6*D*, top row) indicate that the lower dimensional feature may be unrelated to the relocation of the population activity by their preference. Under this condition, the PC1 and PC2 may represent the stimulus feature according to the preferences. We note that the presented information for the best item on PC3 remains unclear and further examine these properties in the Optimal response analysis subsection.

### Quantitative comparisons of two neural population dynamics

**Figure 8.**
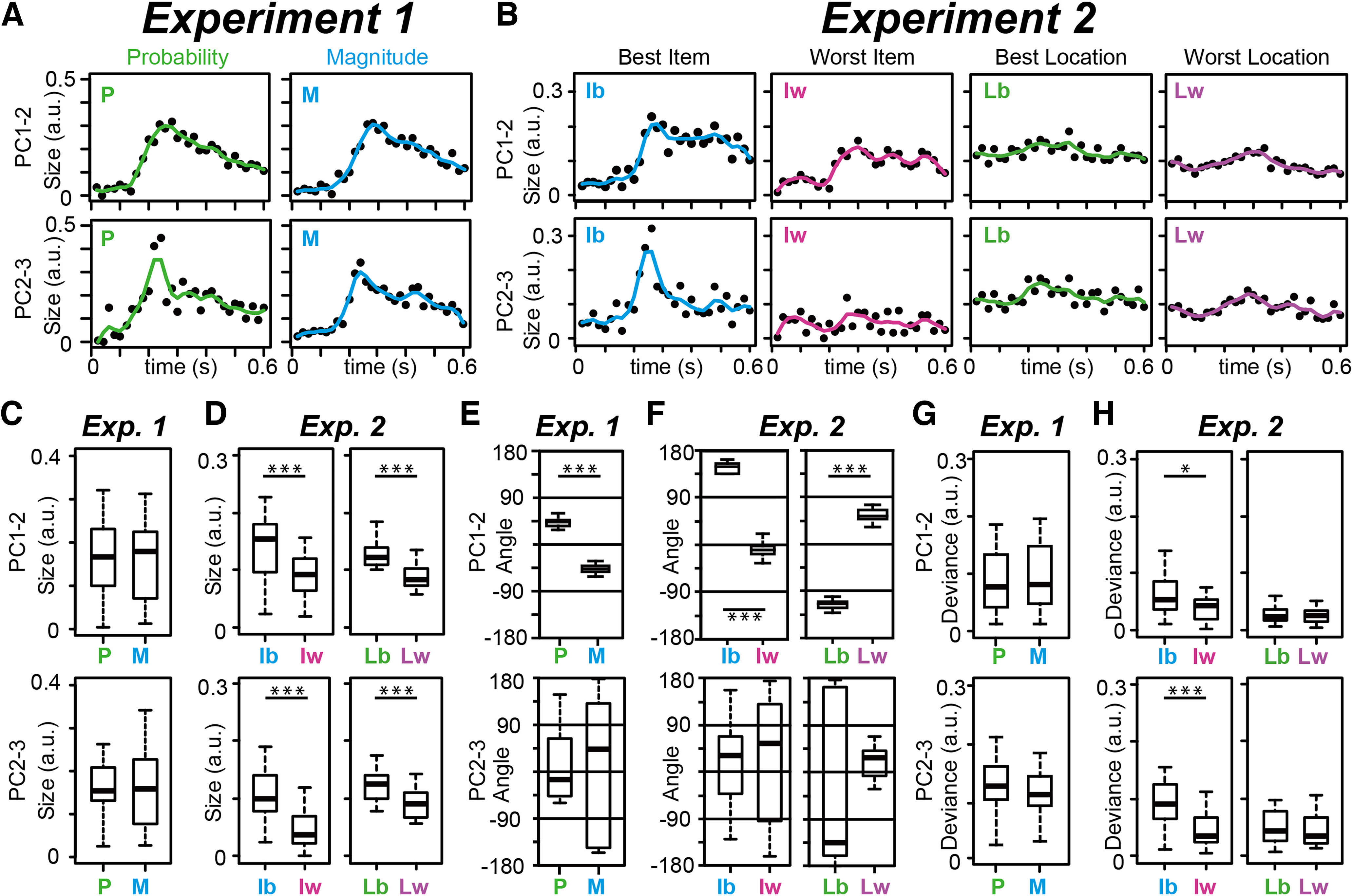
Quantitative evaluations of eigenvector properties in the cOFC and HPC populations. ***A***, Time series of vector size estimated in the cOFC population for P and M of rewards. Vector sizes are estimated in the PC1–PC2 plane (top) and PC2–PC3 plane (bottom), respectively. a.u., Arbitrary unit. The solid-colored lines indicate interpolated lines using a cubic spline function to provide a resolution of 0.005 s. ***B***, Time series of vector size estimated in the HPC population for the best and worst items. ***C***, Boxplots of vector size estimated in the cOFC population for probability and magnitude of rewards. ***D***, Boxplots of vector size in the HPC population for the best and worst items and locations. ***E*, *F***, Boxplots of vector angle estimated in the cOFC (***E***) and HPC (***F***) populations. ***G***, ***H***, Boxplots of vector deviance from the mean estimated in the cOFC (***G***) and HPC (***H***) populations. In ***C–H***, data after 0.1 s are used. *p < 0.05, ***p < 0.001.

To quantitatively examine and compare these neural population structures using geometry, we compared the properties of the eigenvectors by estimating the vector size, angle, and deviance in each neural population (Fig. 8). We analyzed rank-ordered HPC data shown in Figure 7 as well as cOFC data shown in Figure 5. For the rank-ordered data, we evaluated the best and worst conditions as the typically used conditions in conventional rate-coding analyses.

First, the vector size evaluation provided clear time-dependent structures in both cOFC and HPC populations for probability and magnitude (Fig. 8*A*) and for the best and worst items (Fig. 8*B*). Such time-dependent changes were not clearly observed in the eigenvectors for the best and worst locations (Fig. 8*B*, right and second to right columns), because location information already has been provided to the monkeys before the samples appeared. The vector sizes during the period 0.1–0.6 s after the onset of the lottery cue were not significantly different between the two continuous parameters, probability and magnitude of rewards (Fig. 8*C*; Wilcoxon rank-sum test; PC1 to PC2: *n* = 52, df = 51, W = 330, *p *=* *0.892; PC2 to PC3; *n* = 52, df = 51, W = 341, *p *=* *0.964). In contrast, the vector sizes during the period 0.1–0.6 s after the onset of the sample significantly differed between the best and worst items (Fig. 8*D*; Wilcoxon signed-rank test; PC1 to PC2, item: *n* = 52, df = 51, W = 502, *p *=* *0.002; PC2 to PC3, item: *n* = 52, df = 51, W = 588, *p *<* *0.001; PC1 to PC2, location: *n* = 52, df = 51, W = 600, *p *<* *0.001; PC2 to PC3, location: *n* = 52, df = 51, W = 542, *p *< 0.001). This is because the regression coefficients for the best item were considerably different from their mean neural modulation (Fig. 3*J*, *x*-axis) in contrast to those for the worst item (*y*-axis). Thus, the vector sizes captured temporal changes in neural modulation at the population level, which is consistent with the results obtained from the rate-coding analyses (Fig. 3).

The analyses of vector angles showed that all eigenvectors were stable in both populations in the top two dimensions (Fig. 8*E*,*F*, top; Wilcoxon rank-sum test; cOFC, PC1 to PC2: *n* = 52, df = 51, W = 62, *p *<* *0.001; HPC, PC1 to PC2, item: *n* = 52, df = 51, W = 520, *p *<* *0.001; HPC, PC1 to PC2, location: *n* = 52, df = 51, W = 0, *p *<* *0.001). The angles in the PC2–PC3 plane were not stable (Fig. 8*E*,*F*, bottom; Wilcoxon rank-sum test; cOFC, PC2 to PC3: *n* = 52, df = 51, W = 343, *p *=* *0.935; HPC, PC2 to PC3, item: *n* = 52, df = 51, W = 321, *p *=* *0.765; HPC, PC2 to PC3, location: *n* = 52, df = 51, W = 312, *p *=* *0.643). Both neural populations showed some vector deviance (<0.1), with some statistical differences (Fig. 8*G*,*H*; Wilcoxon rank-sum test; cOFC, PC1 to PC2: *n* = 52, df = 51, W = 361, *p *=* *0.683; cOFC, PC2 to PC3: *n* = 52, df = 51, W = 300, *p *=* *0.496; HPC, PC1 to PC2, item: *n* = 52, df = 51, W = 459, *p *=* *0.027; HPC, PC2 to PC3, item: *n* = 52, df = 51, W = 581, *p *<* *0.001; HPC, PC1 to PC2, location: *n* = 52, df = 51, W = 352, *p *=* *0.807; HPC, PC2 to PC3, location: *n* = 52, df = 51, W = 384, *p *=* *0.408).

Collectively, our state-space analysis in the regression subspace described neural modulation dynamics in the cOFC and HPC during two different cognitive tasks composed of continuous and categorical parameters. These dynamic structures, evaluated qualitatively (Figs. 5-7) and quantitatively (Fig. 8), reflected the neural modulation properties described by the conventional rate-coding analyses (Fig. 3). The straight dynamics observed in both cOFC and HPC populations were captured by a combination of changes in vector size and stable vector angle across time, which cannot be captured by the conventional rate-coding framework.

### Optimal-stimulus response analysis in the dynamic model

The rate-coding model typically analyzes optimal and nonoptimal responses of neurons at the population level, which was used to evaluate the extent of neural modulations without dynamics. For the last comparison between the rate-coding and dynamic models, we described neural population dynamics for optimal/nonoptimal activity and compared this with the dynamics obtained from the whole activity (Fig. 7). We reconstructed matrix *X* of the HPC population by extracting the best and worst conditions for item and location, whereas the regression matrix from the other conditions, second to fifth preferred items and second to third preferred locations, were removed. The regression matrix was reduced from the original large size, total *X*, of size *N*_(590)_ × *M*_(10 × 30)_ to *N*_(590)_ × *M*_(4 × 30)_.

In this optimal and nonoptimal regression matrix, PCA captured a greater percentage variance (Extended Data Fig. 7-1*A*, ∼16% of the variance explained by PC1 and PC2). Similar to the original matrix, straight dynamics were observed (Extended Data Fig. 7-1*B*, left column) with a slight difference in vector size between the best and worst items. The PCs appeared to be rotated at an angle of ∼135° from the original on the PC1–PC2 plane (Fig. 7*C*, top row, Extended Data Fig. 7-1*B*, top row). The percentage variance explained by PCA clearly differed from those in the shuffled conditions for the top three PCs (Extended Data Fig. 7-1*C*, circles), and they significantly differed from the shuffled control even in condition 2 (Extended Data Figs. 7-1*C*, 6–1*B*, middle, boxplot, circles, *p *<* *0.05), suggesting that some neural population structures in higher dimensions might be removed in this selected matrix. Thus, the straight dynamics in the lower dimension were clearly derived from this restricted optimal and nonoptimal activity, which represents the main neural modulations in the neural population.

We note that in this optimal response analysis, the first PC represented location information in a stable manner (Extended Data Fig. 7-1*B*, right column), whereas the second and third PCs represented item information (Extended Data Fig. 7-1*B*, left bottom), in contrast to the preference ordering analysis with the full regression matrix (Fig. 7*C*, left bottom). We also note that this reduction of the regression matrix caused relocations of whole vectors because the entire structure of the matrix largely changed.

To summarize, using two standard continuous and categorical task parameters we found stable evolutions of neural modulation structures as the straight geometry over a relatively short period, 0.6 s, when the monkeys perceived the visual items. We incorporated the activity preference analysis used in the rate-coding model and showed that the straight geometry was derived from the preferred/nonpreferred activity modulations. While the tasks and examined brain regions were different in the two neural populations, these straight geometries with a unidimensional feature of neural modulations indicated that the modulation structures by the task parameters retained similarity across time in the lower-dimensional subspace (i.e., similarity in mathematics).

## Discussion

### Temporal structures of neural modulations in the regression subspace

In the present study, we developed a state-space analysis in the regression subspace for categorical task parameters and captured neural modulation dynamics for item and location, as an extension of our previous study (Yamada et al., 2021). Thus, our analysis covers all types of task parameters typically used in the rate-coding model, continuous and categorical. We achieved the following two goals in this article: (1) we newly developed the state-space analysis in the regression subspace for the categorical task parameters; and (2) we found that the HPC neural population exhibited straight dynamics. Fair comparisons of neural modulation dynamics of the two parameters (Figs. 5-8) indicated that straight dynamics observed at the lower dimension exhibited a gradual development (Fig. 8*A*,*B*) and stable composition of the neural modulation structures at different angles (Fig. 8*E*,*F*, top). Thus, we conclude that the neural population structures obtained from different brain regions using different behavioral tasks were similarly stable in terms of geometric changes.

To bridge the rate-coding and dynamic models, we incorporated a standard rate-coding approach into the dynamic analysis, such as activity preference analysis in the categorical task parameters (Fig. 7). We also performed the additional optimal/nonoptimal response analysis using the best and worst conditions for item and location in line with the rate-coding analysis. These analyses showed that straight dynamics were derived from the optimal and nonoptimal activity of neurons (Fig. 7*C*, Extended Data Fig. 7-1*B*). In the rate-coding analysis, the time course of neural modulation by item and location was observed in the percentages of modulated neurons (Fig. 3*I*) and in the magnitudes of regression coefficients (Fig. 3*J*). In the dynamic analysis, these characteristics were observed on the time course of the vector size (Fig. 8*A*,*B*), while the neural modulation structures were evaluated in terms of their similarity across time. Thus, the classical rate-coding analysis was well incorporated into the dynamic analyses, which specifically captured their neural population geometries.

In the rate-coding analysis, the extent of neural modulations at the level of the population is analyzed by relocating their activity using activity preference, such as the preferred direction of movement, preferred location, or preferred stimulus. In our dynamic analysis incorporating the activity preference approach (Fig. 7), we have observed similar structures in neural dynamics between preference-ordered (Fig. 7) and nonordered (Fig. 6) neural population activity at the lower dimensional. What does this comparison provide as an aspect for understanding information processing? The neural population structures relocated by the preference ordering should affect the neural population dynamics, although the entire PC1–PC2 spaces were similar (Figs. 6*D*, top row, 8*C*, top row) and third PC changed (Fig. 7*C*, left bottom). Further, the elimination of the population activity except for the best and worst activity changed the trajectory at the lower dimensional spaces, but the straight geometry remained apparent (Extended Data Fig. 7-1). These observations indicated that the straight unidimensional feature in their dynamics at the lower dimension is derived from the activity relationship between optimal and nonoptimal conditions, while the whole allocation of geometry depends on the entire structure of neural population activity. The extent of neural modulation might be represented in the lower dimensional neural modulation space after the preference ordering.

Both cOFC and HPC neural populations consisted of separately recorded single-neuron activities after visual stimulus presentations, and we extracted both neural modulation dynamics using an original state-space analysis. These straight geometries with unidimensional features of neural modulations indicated that modulation structures by task parameter remained similar across time (Fig. 3*E*,*J*). We would like to caution researchers against using nonmodulated task parameters in PCA (i.e., task parameters that show minimal neural modulation), as the PCs derived in such cases were less biologically meaningful (Björklund, 2019). Our simple extractions of neural modulation dynamics indicate that all types of data can be easily reanalyzed and evaluated to find the trajectory geometry of neural modulations, which can be compared with pre-existing rate-coding evidence.

### TDR and dPCA analyses

Two predominant dynamics analyses for task-related parameters exist, TDR and dPCA. The dPCA was aimed at detecting latent components related to discrete task parameters (Brendel et al., 2011; Kobak et al., 2016) by decomposing neural population data. In contrast, TDR was aimed at identifying the encoding axis of the task parameters in a multidimensional neural space (Mante et al., 2013; Aoi et al., 2020) by incorporating linear regression into neural population dynamics with respect to task parameters. Both methods are the state of the art and shed light on the link between the rate-coding and dynamic models by visualizing complex datasets with multiple task parameters. Moreover, Elsayed and Cunningham (2017) compared the neural population responses with surrogate data that simultaneously preserve the temporal correlation of discharge rates, signal correlations across neurons, and tuning to the experimental parameters of the task.

**Figure 9.**
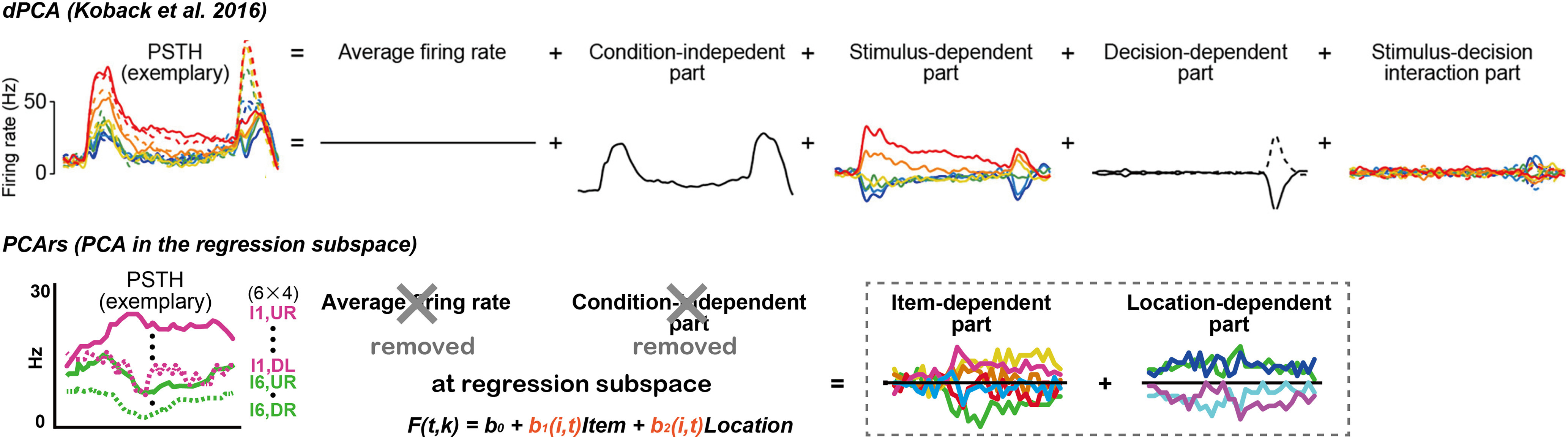
Thematic depiction of the difference between dPCA and PCArs. Illustration of the procedure to construct the neural population dynamics by dPCA and ours (PCArs). dPCA decomposes the neural activity into the dynamics as a linear summation of the multiple components for categorical variables (Kobak et al., 2016). Our method first projects the neural population dynamics into a regression subspace that removes the activity change other than the neural modulations by task parameters and demonstrates the modulation dynamics. dPCA figures are represented in the study by Kobak et al. (2016, their Fig. 8). PSTH indicates peristimulus time histogram.

These procedures have several limitations when precisely applied to data and they require multiple mathematical steps for the user to apply these elaborate methods. In contrast, our method is simpler and more intuitive and directly extracts the temporal structures of neural modulations by the task parameters. If we compare the dPCA and our PCA in the regression subspace (PCArs), the dPCA decomposes all neural signals (Fig. 9, top row), but PCArs projects the neuronal activity into the regression subspace at the population level by removing the average activity in each moment (Fig. 9, bottom row). Our methods are similar to the dPCA, but not the same. For example, we did not include the interaction component since the state-space analysis assumes a linear system. PCArs only focuses on neural dynamics in the subspace and requires only two steps (Fig. 4, middle, bottom rows) and minimal assumptions (orthogonality in the task design). Our PCArs is beneficial in terms of being simple and intuitive as it is an extension of the conventional rate-coding approach. Thus, our state-space analysis provides researchers with a significant advantage to find modulation dynamics in lower-dimensional subspaces for pre-existing datasets.

Another analysis of regression subspace, TDR, has been performed in a limited number of studies (Mante et al., 2013; Aoi et al., 2020). These studies aimed to detect the regression subspace within a neural structure at a constant time point (Mante et al., 2013). The detected modulation axis was assumed to be projected orthogonally and sometimes stably through a task trial (Aoi et al., 2020). In contrast, we directly detected neural population dynamics in the regression subspace, which was a straight dynamic for both continuous and categorical task parameters. Because the axis of neural modulation remains stable in these straight dynamics (Figs. 5, 6), our results support assumptions regarding the stability of the regression subspace, which were not examined in previous studies.

### Two different types of task parameter yield comparable dynamics in regression subspaces

In our state-space analysis, neural population activity was projected onto the regression subspace, reflecting the across-trial variance caused by task-related parameters at the population level. In this step, both continuous and categorical task parameters were reliably used within the framework of the general linear model. However, it was reliably performed with one limitation; the conditions in any parameter would be orthogonalized as the experimental design (Grafen and Hails, 2002). For the continuous task parameters in Exp. 1, the 10 × 10 conditions for probability and magnitude were orthogonalized. In the case of categorical task parameters in Exp. 2, 6 × 4 conditions for item and location were orthogonalized. For both experiments, the number of recorded trials for each neuron was almost the same for all conditions. Thus, the projections of neural activity to the regression subspace were nearly orthogonal.

In the linear system assumed here, the concept of orthogonality is critical in terms of statistics and to avoid skewed projection of neural activity into the regression subspace, which is part of the entire neural activity, reflecting activity modulation by the task parameters of interest (Fig. 9). However, one concern for the comparison between the two distinctive datasets is observed in the percentages of the variance explained by the PCA (Figs. 5*A*, 6*A*). The smaller percentages of the variance explained by the model in the HPC might be because of the larger matrix size. While the dynamics observed in the HPC population were not meaningless, the descriptive ability at the lower dimension is different from that in the cOFC.

In this study, we evaluated neural modulation dynamics in terms of vector size, angle, and deviance in the regression subspace (Extended Data Fig. 4-1) that composes possible trajectory geometries. The combination of vector size and angle describes our straight geometry (i.e., constant angle), whereas deviance also reflects vector stability over time. The straight geometry indicated that modulation structures by the task parameters kept similarity across time in the multidimensional neural subspace. If neural modulations are completely stable across time (i.e., all vectors are the same across time), then all three parameters become very stable (i.e., congruence in mathematics: constant vector angle, constant vector size, and very small deviance). Thus, our analysis of vector properties (Fig. 8) can quantitatively evaluate trajectory geometry in neural modulations.

### Stable and fluctuating signals in neural modulation dynamics

In this study, we observed stable straight neural modulation dynamics in both cOFC and HPC populations, a divergent excursion with a straight trajectory in a subspace plane. Although these tasks were designed using different task parameter types, both brain regions showed similar stable-modulation structures during the visual perception (Figs. 5-8). One possible explanation for these two brain regions exhibiting stable modulation dynamics is that they may share similar information processing when accessing memories for expected values as a combination of probability and magnitude (as reflected in Exp. 1) and the association between stimulus and position for future decisions (Exp. 2). Access to some memories may stabilize the neural population activity. More stable structures have been observed in the dorsolateral prefrontal cortex during a typical working memory task (Murray et al., 2017), although this activity was highly stable through memory maintenance. Comparisons of the stability in neural modulations would not be easy using conventional analyses based on the rate-coding model. Another important aspect is whether behavioral change (or policy) affects trajectory geometry, since neurons in the dorsolateral prefrontal cortex have shown unstable dynamics during a choice task (Mante et al., 2013). In our experiments, this test was not performed because of the no-choice data in Exp. 1.

In our previous study, fluctuating neural population signals were observed in the dorsal striatum (DS) and medial OFC (mOFC) because of signal instability or weakness (Yamada et al., 2021, their Fig. 5A,B; Imaizumi et al., 2022). As the signal carried by the mOFC population was weak (Yamada et al., 2021, their Fig. 8, bottom row), eigenvector fluctuation in the mOFC population reflected the weak signal modulations by the probability and magnitude of rewards. In this case, moment-by-moment vector fluctuations were observed because there was no clear neural modulation structure in the mOFC population. Conversely, the fluctuating DS signal seemed to reflect the functional role of the DS neural population in detecting and integrating the probability and magnitude of rewards related to the control of some actions (Balleine et al., 2007; Enomoto et al., 2020; Inokawa et al., 2020). In the DS population, structural changes in eigenvectors occurred over time (Yamada et al., 2021, their Fig. 8). Future studies are required to explore neural modulation geometry to elucidate how neural circuitry operates and computes (Ebitz and Hayden, 2021; Humphries, 2021).

### Conclusions

Rate-coding models have provided mounting evidence that neural modulation is associated with task parameters in many regions of the brain (e.g., movement direction, muscle force, place, and reward value). A recently developed dynamic model approach appears promising to account for the different aspects of neural information processing. However, its relationship with rate-coding models remains unclear. Although some studies have sought a connection between these two advances (Mante et al., 2013; Chen and Stuphorn, 2015; Kobak et al., 2016; Murray et al., 2017; Aoi et al., 2020), direct comparisons are necessary to link the two analytical results. Our results allow us to consider whether neural modulation dynamics observed in neural population ensemble activities are compatible with rate-coding models. Specifically, trajectory geometries provided temporal dynamics of neural modulation that could bring about new insights into neural processing and conceptual advances, such as straight geometry with a unidimensional feature. Thus, our simple approach encourages research aimed at incorporating traditional rate-coding models into dynamic systems as neural modulation dynamics.

## Synthesis

Reviewing Editor: Mark Laubach, American University Decisions are customarily a result of the Reviewing Editor and the peer reviewers coming together and discussing their recommendations until a consensus is reached. When revisions are invited, a fact-based synthesis statement explaining their decision and outlining what is needed to prepare a revision will be listed below. The following reviewer(s) agreed to reveal their identity: NONE.

Both reviewers appreciated the revisions that were made. One reviewer was satisfied with the manuscript. The other asked for minor revisions (no new data analysis). Please see this reviewer’s comments below. Please revise your paper accordingly and I will review the changes before making a final decision.

Thank you for sending your work to eNeuro!

Reviewer comments:

Thank you authors for all efforts to answer questions from previous comments. I appreciated that authors added schematic depictions in current Figure 1, graphical methods in current Figure 4, and shuffle control in current Extended Data Figure 6-1 for reference. The remaining question I have below is related to my third question from previous comments. It would be great to learn whether the results found in both rate-coding and regression subspace models could be integrated and provide broad insights.

Major comments:

1) Around 40% units in either cOFC (36.3%, Figure. 3C) and hippocampus (45%, Figure. 3H) populations do not selectively respond to task variables from conventional rate-coding analyses. Based on this, I would expect that there is no correlation when plotting regression coefficients (b1 and b2) included only non-significant units (like current Figure 3E and 3J, but without selective units). Therefore, I wonder whether or not authors have tried to recruit only selective units into the analysis of two-steps dynamical model? This concern is about uncorrelated data points may lead to biased eigenvectors and eigenvalues after applied PCA (A simple example is explained here: https://doi.org/10.1111/evo.13835). If this concern is reasonable, could authors take it into account and find a way to make corrections in the two-step dynamical model? This correction may take both rate-coding and dynamical models into account and improve the results in two experiments, especially in experiment 2. If this concern has no influence on the current results, please explain why.

Minor comments:

1) To include the main feature, regression subspace, of your 2-steps dynamical model in the current Figure 1, could authors add a sample diagram as the second step in between neural modulation and PCA?

2) To better understand the distributions of regression coefficients (b1 and b2) in Figure 3E and 3J, could authors color-label significant neurons by task variables (e.g. Figure 3C ad 3E) and count how many significant units in each quadrant?

3) Typo in Figure 9: the title of exemplary plot in the bottom of Figure 9 should be PSTH.
