# Supplementary figures and images for: Stable Neural Population Dynamics in the Regression Subspace for Continuous and Categorical Task Parameters in Monkeys
Source: eNeuro. 2023 Jul 7;10(7):ENEURO.0016-23.2023. doi: 10.1523/ENEURO.0016-23.2023 (PMC10337837; doi:10.1523/ENEURO.0016-23.2023)

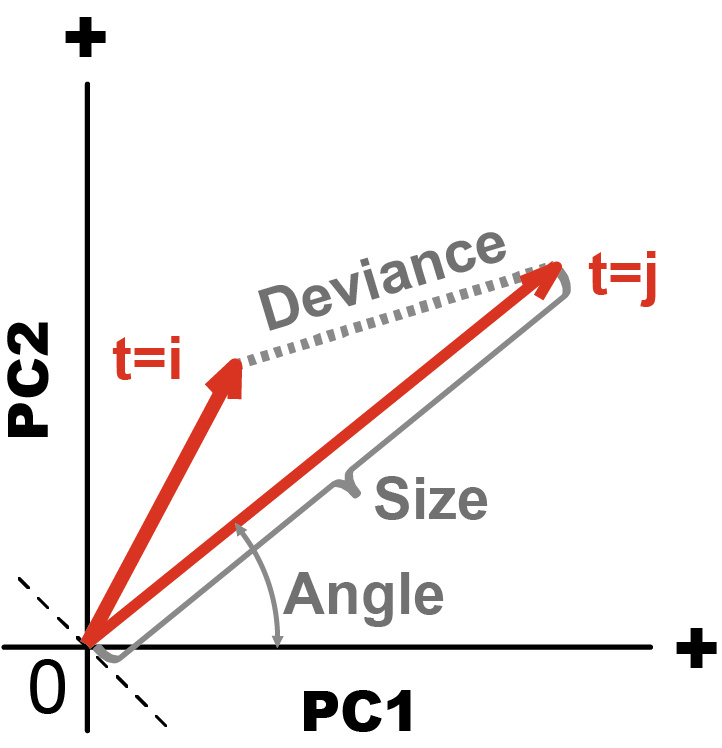

Supplement: Figure 4-1 — Schematic depictions of eigenvector evaluations. Characteristics of the eigenvectors evaluated quantitatively. Angle, vector angle from the horizontal axis obtained from –180° to 180°; Size, eigenvector length; Deviance, difference between vectors. Download Figure 4-1, file. [file enu-eN-NWR-0016-23-s02.tif]

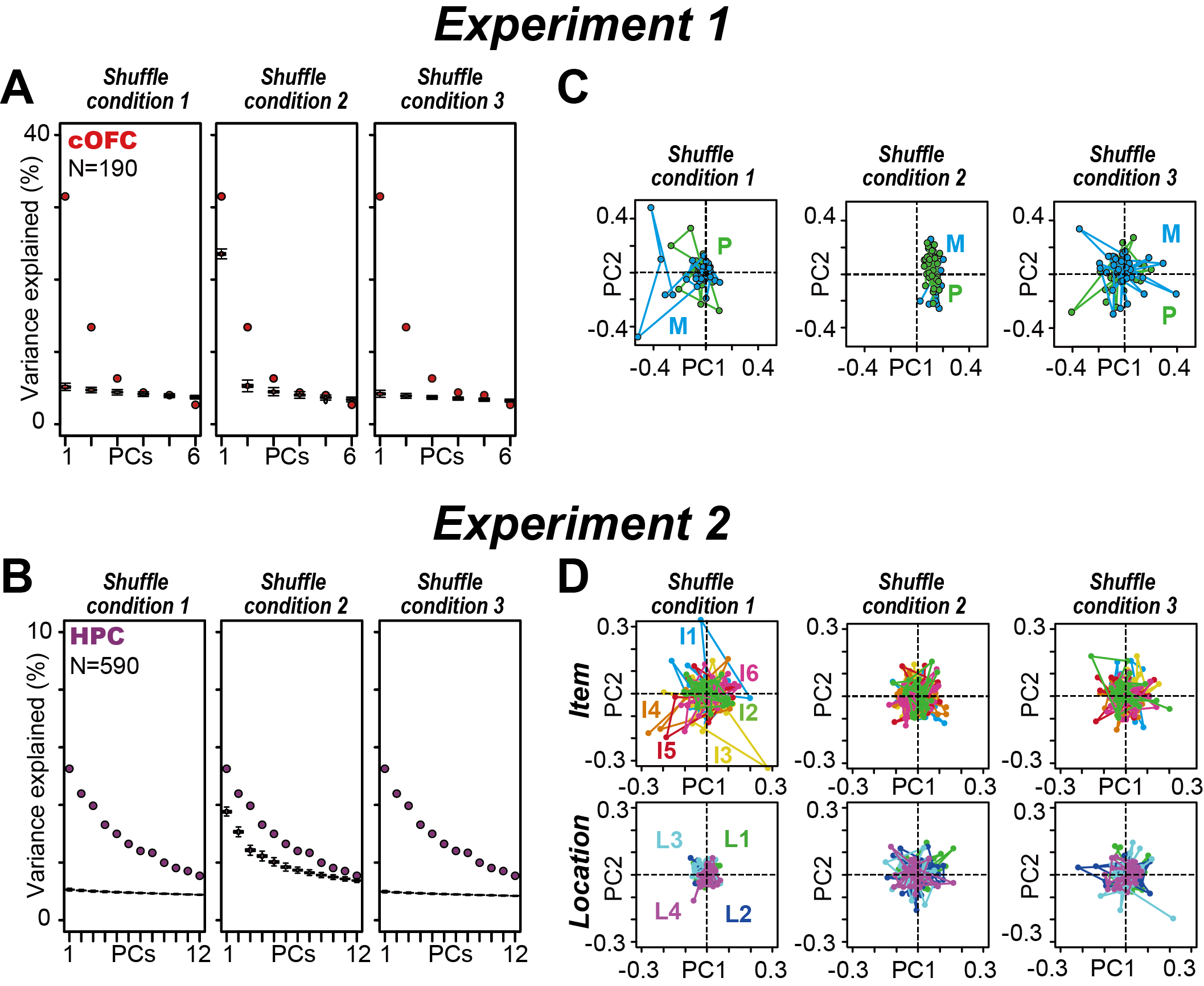

Supplement: Figure 6-1 — Explained variances by PCA in shuffled controls. A, A boxplot of explained variances by PCA for PC1 to PC6 for the cOFC population under the three shuffled conditions (for details, see Materials and Methods). The plot is not cumulative. The boxplot was made with 1000 repeats of the shuffle in each condition. B, A boxplot of explained variances by PCA for PC1 to PC12 for the HPC population. In A and B, the colored circles indicate variances explained by PCA in each neural population without the shuffles. C, Examples of a series of eigenvectors for PC1 to PC2, plotted in the cOFC population under the three shuffle conditions. D, Examples of a series of eigenvectors for PC1 to PC2, plotted in the HPC population under the three shuffle conditions. Download Figure 6-1, file. [file enu-eN-NWR-0016-23-s03.tif]

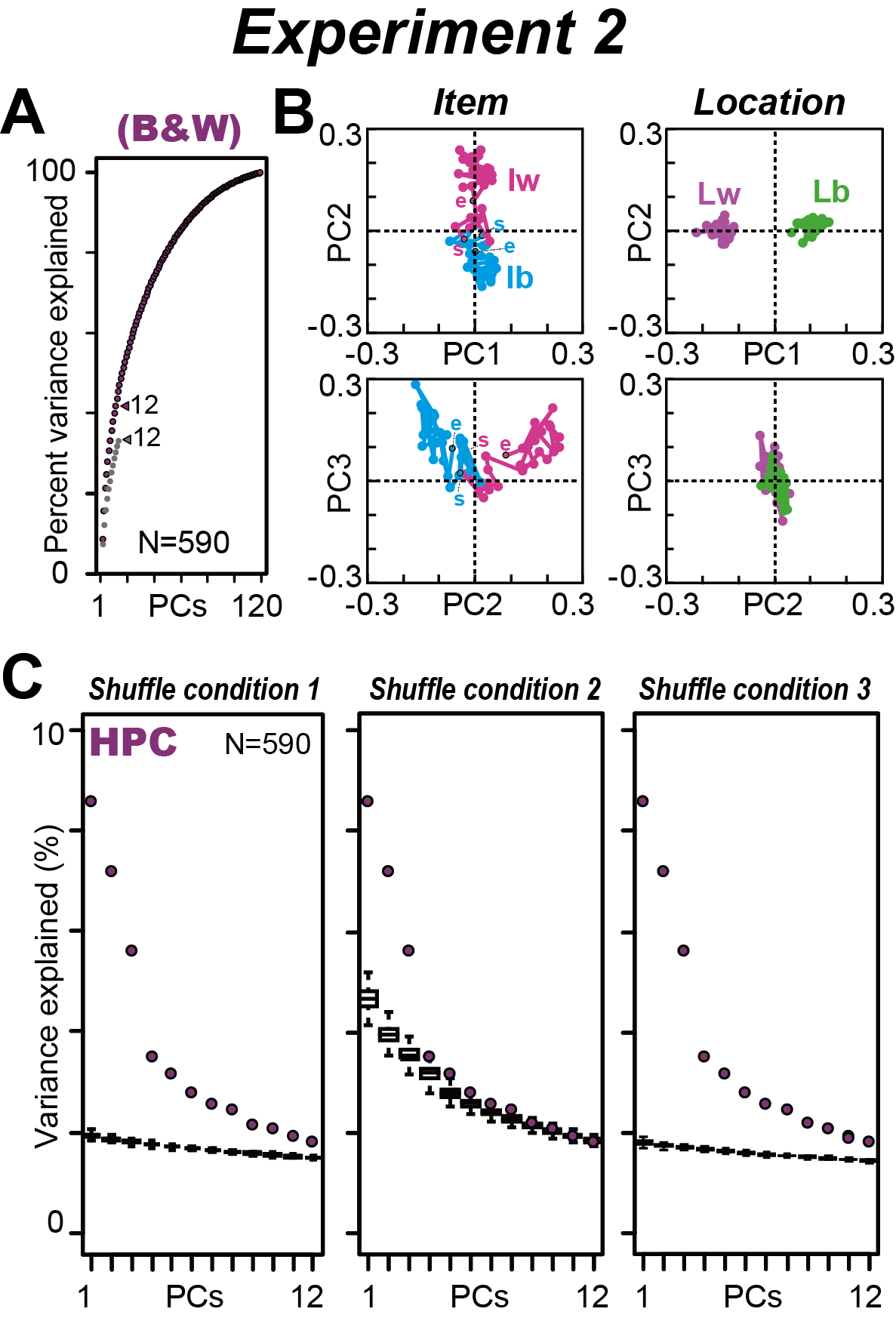

Supplement: Figure 7-1 — Optimal response analysis in the HPC population. A, Cumulative variance explained by PCA in the HPC population when the best and worst conditions for item and location were used for the regression subspace. The gray dots indicate the percentage variance explained by PCA upon using the full matrix. The first 12 PCs are shown. B, Time series of the eigenvectors for PC1 to PC3 when the best and worst items and locations were used. Ib and Iw indicate the best and worst items, respectively. Lb and Lw indicate the best and worst locations, respectively. s and e indicate the start and end of the time series of vectors, respectively. C, A boxplot of explained variances by PCA for PC1 to PC12 under the three shuffled conditions (for details, see Materials and Methods). The plot is not cumulative. The boxplot was made with 1000 repeats of the shuffle in each condition. The colored circles indicate the variances explained by PCA in the HPC population without the shuffles. Download Figure 7-1, file. [file enu-eN-NWR-0016-23-s04.tif]
